# Mapping the splicing landscape of the human immune system

**DOI:** 10.3389/fimmu.2023.1116392

**Published:** 2023-08-30

**Authors:** Hadas Ner-Gaon, Ronnie Peleg, Roi Gazit, Anat Reiner-Benaim, Tal Shay

**Affiliations:** ^1^ Department of Life Sciences, Ben-Gurion University of the Negev, Beer-Sheva, Israel; ^2^ The Shraga Segal Department of Microbiology, Immunology and Genetics, Faculty of Health Sciences, Ben-Gurion University of the Negev, Beer-Sheva, Israel; ^3^ Department of Epidemiology, Biostatistics and Community Health Sciences, School of Public Health, Faculty of Health Sciences, Ben-Gurion University of the Negev, Beer-Sheva, Israel

**Keywords:** alternative splicing, differential splicing, alternative promoters, immune system, immune related diseases, sepsis

## Abstract

Most human genes code for more than one transcript. Different ratios of transcripts of the same gene can be found in different cell types or states, indicating differential use of transcription start sites or differential splicing. Such differential transcript use (DTUs) events provide an additional layer of regulation and protein diversity. With the exceptions of PTPRC and CIITA, there are very few reported cases of DTU events in the immune system. To rigorously map DTUs between different human immune cell types, we leveraged four publicly available RNA sequencing datasets. We identified 282 DTU events between five human healthy immune cell types that appear in at least two datasets. The patterns of the DTU events were mostly cell-type-specific or lineage-specific, in the context of the five cell types tested. DTUs correlated with the expression pattern of potential regulators, namely, splicing factors and transcription factors. Of the several immune related conditions studied, only sepsis affected the splicing of more than a few genes and only in innate immune cells. Taken together, we map the DTUs landscape in human peripheral blood immune cell types, and present hundreds of genes whose transcript use changes between cell types or upon activation.

## Introduction

Transcript diversity is created by several mechanisms, including alternative transcription start sites (TSS), alternative splicing, and alternative polyadenylation sites. Multiple TSSs are found in at least 58% of human genes (1), and these TSSs transcribe different pre-mRNAs and are potentially regulated by different transcription factors (TFs). Alternative splicing results in multiple transcripts from the same pre-mRNA, which are potentially regulated by different splicing factors (SFs). Multiple polyadenylation sites, which exist in most eukaryotic genes, create 3’ untranslated regions of different lengths (2, 3). As a result of one or more of the above mechanisms, more than 95% of human genes express more than one transcript (4, 5). These transcripts differ in their RNA sequences, which may affect protein sequence, RNA stability, RNA localization and RNA regulation. Indeed, aberrant transcripts play important roles in various diseases, including cancers and autoimmune and infectious diseases (6–8). Genome-wide studies of known transcript diversity have been facilitated by the development of exon microarrays, and the subsequent development of RNA sequencing (RNA-seq) has enabled those studies to be extended to transcripts that had not previously been annotated. These studies notwithstanding, the abundance and importance of transcript diversity during immune differentiation and activation is only just beginning to be uncovered (9–12). A new study identified 55 genes with transcript-level differential expression between seven human immune cell types from twelve healthy donors, and 27 genes with transcript-level differential expression between monocytes, macrophages, and LPS activated macrophages (13).

Conditions that change splicing may affect multiple genes simultaneously. In each cell type or condition, the change to each alternative splicing type may be different; for example, neutrophils are characterized by higher levels of intron retention than other immune cell types (14, 15). The response of human innate immune cells to pathogens is accompanied by enhanced transcription of minor and non-coding isoforms, noisy splicing, enhanced inclusion of skipped exons, and the use of more upstream polyadenylation sites (16–18). Additionally, infection may induce alternative splicing (19). For example, following inflammation, the dominant class of alternative isoform usage in human and murine macrophages is alternative first exon usage (20). Differentiation may also induce splicing changes; for example, a comparison of 11 murine immune cell types revealed that many differential splicing events were linked to lineage differentiation (21). Despite these genome-wide reports, only a few cases of differential transcript use (DTUs) events of specific genes in the immune system have been thoroughly studied, namely, those in immunoglobulins (22), CIITA (23), and PTPRC/Cd45 (24). In addition, SMPD1 is known to be differentially spliced in human sepsis (25). However, the functional extent of transcript diversity in immune cells and the changes in the use ratio of the different transcripts in different human immune cell types in health and immune-related conditions remain to be elucidated.

A better understanding of DTU events and their regulation in the human immune system requires a transcriptome-level comparison of the different immune cell types, which is yet to be done. In this genome-wide study, we set out to identify DTU events in healthy immune cell types in health and immune related conditions. We found 282 genes with DTUs between different human immune cell types in four datasets (**Figure S1A**). The correlation between the pattern of DTU events and the expression of SFs and TFs suggests a regulatory model for DTU in the immune system. When comparing cells related to immune conditions to healthy cells of the same type, we found 67 DTUs. We also found four genes with DTU events between cells before and after treatment for multiple sclerosis (MS). Among different immune-related conditions, namely amyotrophic lateral sclerosis (ALS), MS (pre and post treatment), type 1 diabetes, and sepsis, the effect of sepsis on splicing was the most marked. A deeper understanding of DTU regulation in each cell type and for different immune-related conditions could contribute to better control of the immune response and hence to improved treatment of patients in whom the immune system damages tissues, as in sepsis.

## Materials and methods

### Datasets

To identify differential splicing in the human immune system, a search was done in GEO site (https://www.ncbi.nlm.nih.gov/geo/) to find all the publicly available RNA-seq datasets that profiled at least five healthy human immune cell types: B cells, T cells, natural killer (NK) cells, monocytes and neutrophils, and include at least seven samples. Specifically the search query: “b-lymphocytes”[MeSH Terms] AND “t-lymphocytes”[MeSH Terms] AND “killer cells, natural”[MeSH Terms] AND “monocytes”[MeSH Terms] AND “neutrophils”[MeSH Terms] AND “Homo sapiens”[porgn] AND (“7”[n_samples]: “100000”[n_samples]) AND “Expression profiling by high throughput sequencing”[Filter] was used. That query on 23/4/2023 returned 12 results, out of which four include all the cell types and at least two repeats per cell type and are not single cell RNA-seq. The datasets are: GSE107011 (26), GSE115736 (27), GSE64655 (28) and GSE60424 (29). As CD4 and CD8 T cells are not profiled in GSE64655, which profiles T cells, they were grouped together for the purpose of comparing between datasets (**Table 1**).

**Table 1:** Structure of the datasets and number of repeats.

Cell type	GSE64655	GSE60424	GSE107011	GSE115736
**B**	2	4	4	5
**CD4 T**		4	4	5
**CD8 T**		4	4	5
**T**	2			
**NK**	2	4	4	5
**Neutrophils**	2	4	4	3
**Monocytes**	2	4	4	5

In dataset GSE60424 cells were also profiled from immune-related conditions. The immune related conditions are ALS, type 1 diabetes, MS and sepsis (29) (**Table 2**). All the donors, with the exception of one, were < 60 years old (**Table S1**).

**Table 2:** Immune related conditions in GSE60424 and number of repeats.

Cell type	Healthy	ALS	Sepsis	Diabetes	MS-pre treatment	MS-post treatment
B	4	3	3	4	3	3
CD4 T	4	3	3	4	3	3
CD8 T	4	3	3	4	3	3
NK	4	3	2	2	1	2
Neutrophils	4	3	3	4	3	3
Monocytes	4	3	3	4	3	3

In the current study, comparisons were conducted for each of the cell types for which there were at least two repeats (except NK in MS pre treatment, **Table 2**). We note that the GSE64655 dataset also profiles control and trivalent inactivated influenza (TIV) vaccine receivers, but we did not compare healthy and TIV cells for that dataset, as differential splicing analysis between those states in each cell type was already reported in the original publication (28), with very few results.

Reads from each dataset were mapped to the *Homo sapiens* genome (hg38) using hisat2 (30) (version 2.0.5). Bam files were sorted and indexed by SAMtools (31) (version 1.9). To evaluate the quality of the data, the flagstat option of SAMtools was used. In all datasets, there were no samples with less than one million properly mapped reads.

### Differential splicing analysis

LeafCutter version 0.2.8 (32) was used according to the workflow described in https://davidaknowles.github.io/leafcutter/articles/Usage.html. The *H. sapiens* genome annotation file (GCF_000001405.39_GRCh38.p13_genomic.gff) was downloaded from NCBI https://www.ncbi.nlm.nih.gov/genome/51 and parsed to fit the LeafCutter input format. Only annotation of genes located on chromosomes 1-22, X, and Y was used. LeafCutter assigns junctions to LeafCutter clusters (LCs). Any two junctions in a cluster share a start or an end position. Poorly covered junctions [more than 10 junction spanning reads (JSR) in less than two samples] were removed from the count table. After the removal of the poorly covered junctions, junctions with a JSR count of <10% of the average JSR count in their LCs in all samples were also removed from the count table to minimize false positive results. If this filtering produced LCs that include junctions without a shared position, those LCs were split to two or more LCs that each includes only junctions that share a start position or an end position (**Figures S1B, C**). The LCs that then remained with less than two junctions were removed from the count table.

In the healthy cells analysis, we implemented hierarchical statistical testing and applied a suitable multiple testing adjustment (33) to control the false discovery rate (FDR). First, we tested each LC for differential splicing between multiple conditions. Then, for each significant LC (FDR-adjusted p-value ≤0.05), we compared all pairs of conditions. Due to the hierarchical scheme, the total number of comparisons decreased considerably, thereby shrinking the problem of multiple testing and optimizing the statistical power (34). For the comparison of the results between datasets, a comparison with either CD4 or CD8 T cells that was significant was considered significant with T cells.

The fraction of JSRs of a junction in each LC in each sample is termed the percent spliced in (PSI). Differential splicing is measured in terms of the change in PSI between conditions, ΔPSI. Differential splicing analysis was performed on three sets: (1) between all the different healthy cell types in each dataset separately; (2) for each cell type in the GSE60424 dataset, between healthy cells and cells from each immune-related condition separately; and (3) for each cell type in the GSE60424 dataset, between MS pre-treatment and MS post-treatment. Each comparison produced a ΔPSI value. Max(ΔPSI) was defined as the maximal abs(ΔPSI) value in all comparisons from the same set that gave significant results.

An LC was considered to be differentially spliced (DS-LC) if all the following conditions were satisfied:

1. Only for the healthy cells analysis, differential splicing in the LC was significant, in LeafCutter multiple analysis, with LeafCutter’s p.adjust ≤ 0.05. Default parameters were used, except for –i 2 (min samples per junction), and -g 0 (min samples per group).
2. Differential splicing in the LC was significant, in LeafCutter paired analysis, with LeafCutter’s p.adjust ≤ 0.05. Default parameters were used, except for -M 10 (minimum reads for a junction), -i 2 (min samples per junction), and -g 2 (min samples per group).
3. The LC contained at least two junctions with max(ΔPSI) ≥ 0.1.
4. The LC contained at least one junction with max(ΔPSI) ≥ 0.2.

Clustering and SF/TF correlation was done only on junctions that are shared between at least two datasets. Clustering of SF/TFs and junctions was performed using the Python scipy.cluster.hierarchy.linkage function. Junctions clustering was done with the seuclidean metric and complete method. SF/TF clustering was done with the correlation metric and average method. The number of clusters was set to 10.

LCs that are significantly differentially spliced between one cell type and all other cell types are defined as cell-type-specific in the context of the five cell types tested. LCs that are differentially spliced between the three lymphocytes (B, T and NK cells) and the two myeloid cell types (monocytes and neutrophils) tested are defined as lineage-specific in the context of the five cell types tested.

Sashimi plots were drawn using the ggsashimi program (35). The *H. sapiens* genome annotation file (Homo_sapiens.GRCh38.104.gtf.gz) was downloaded from Ensmbl http://ftp.ensembl.org/pub/release-89/gtf/homo_sapiens/ and parsed to fit the ggsashimi input format.

### Classification of alternative splicing type

Determination of the alternative splicing type was based on the transcriptome annotation file GCF_000001405.39_GRCh38.p13_genomic.gff, downloaded from https://www.ncbi.nlm.nih.gov/genome/51. Each LC could be classified into several alternative splicing types, or none at all. An LC was classified as a skipped exon (SE; **Figure S2A**) if there was a junction that skipped a specific exon, a junction that ended in the start point of that exon and a junction that started at the end point of that exon, and at least two of those junctions had ΔPSI ≥ 0.1. For the remaining alternative splicing types, only junctions with ΔPSI ≥ 0.1 were used. LCs were classified as mutually exclusive exons (MXE; **Figure S2B**) in genes that were mapped to two LCs, where two junctions started at the same point in the upstream LC and two junctions ended at the same point in the downstream LC. LCs were classified as alternative 5’ splice sites (A5SS; **Figure S2C**) if at least two junctions in the LC exited a specific exon at different points and entered the consecutive exon at the same point. LCs were classified as alternative 3’ splice sites (A3SS; **Figure S2D**) if at least two junctions exited a specific exon at the same point and entered the consecutive exon at different points. LCs were classified as alternative first exons (AFE; **Figure S2E**) or alternative last exons (ALE; **Figure S2F**) if at least one junction in the LC connected to the first or last exon, respectively.

### Cell preparation

Peripheral blood cells were isolated from a healthy donor in EDTA tubes. Mononuclear cells enriched over ficoll (Lymphoprep#7851, StemCell™), and washed with FACS media (PBS, 2mM EDTA, 2% serum). Staining used Biologend antibodies: CD14-PrcCy5.5, CD4-Fitc, CD56-Pe, CD8-PeCy7, CD19-Alexa700, CD3e-PacBlue. Sort by AriaII (BD). RNA extracted by Trizol, and Reverse transcription used SuperScript IV (both Invitrogene™, standard manufacturer protocol).

### PCR

The cDNA was diluted to represent 500 cells/µl, 2 µl taken per PCR reaction representing 10^3 cells. Primers used: FYB1ex14for-tgatgaaacagggaaatcagagg; FYB1ex11rev-ATTCCCTCCACCACCAgatg (yielding 283bp product with exon 12, or 145bp product when exon 12 is skipped); CD47ex11for-CGTCTTACTACTCTCCAAATCGG; CD47ex7rev-atgcatggccctcttctgat (yielding 220bp product with exons 9 + 10, or 162bp product when both exons are skipped). The PCR amplification program used was 95°C 3min, 33 cycles: 95°C 7sec, 58°C 30sec, 72°C 30sec; and finished with 72°C 1 min. PCR products were resolved on 2% agarose gel and visualized with Ethidium Bromide.

### Inferring splicing regulation

Potential SFs were defined as all genes with a GO annotation of ‘GO:0008380, RNA splicing’ in https://www.ebi.ac.uk/QuickGO/. Only SFs that were expressed (at least 10 reads in at least one sample in at least two datasets) and were differentially expressed between immune cell types (one-way ANOVA p value ≤ 0.05, implemented by Python scipy.stats.f_oneway function) were used. The Pearson correlation coefficient between SF expression patterns and the PSI patterns of junctions from LCs with significant differential slicing in at least one comparison that was not classified as an alternative first exon and ΔPSI ≥ 0.2 was calculated, using python corr function. Python seaborn.clustermap was used to plot clustered heatmaps. Correlation between the SF-junctions correlation matrix in each two datasets was calculated by the python pearsonr function.

For the calculation of the Pearson correlation coefficient between SF expression patterns in a pair of datasets, the average expression values per cell type were used, where all T cells were averaged together.

### Inferring transcriptional regulation

Potential TFs were defined as all genes with a GO annotation of ‘GO:0009299, mRNA transcription’ in https://www.ebi.ac.uk/QuickGO/. Only TFs that were expressed (at least 10 reads in at least one sample in at least two datasets) and were differentially expressed between immune cell types (one-way ANOVA p value ≤ 0.05, implemented by Python scipy.stats.f_oneway function) were used. The Pearson correlation coefficient between TF expression patterns and the PSI patterns of junctions from LCs with significant differential slicing in at least one comparison that was classified as an alternative first exon and ΔPSI ≥ 0.2 was calculated, using python corr function. Python seaborn.clustermap was used to plot clustered heatmaps. Correlation between the TF-junctions correlation matrix in each two datasets was calculated by the python pearsonr function.

For the calculation of the Pearson correlation coefficient between TF expression patterns in a pair of datasets, the average expression values per cell type were used, where all T cells were averaged together.

## Results

### Differential splicing between healthy immune cell types

We identified DTU events between healthy B cells, T cells, NK cells, monocytes and neutrophils in four human datasets, GSE107011 (26), GSE115736 (27), GSE64655 (28) and GSE60424 (29) (**Tables 1**; **S2**, **Figure S1A**). There are 458 DS-LCs in the GSE60424 dataset (**Table S3**), 224 DS-LCs in the GSE64655 dataset (**Table S4**), 196 DS-LCs in the GSE107011 dataset (**Table S5**), and 744 DS-LCs in the GSE115736 dataset (**Table S6**). When comparing the four datasets, we discuss genes or junctions, as the LeafCutter clusters are defined separately for each dataset. There are 725 genes that contain a DTU in only one dataset, 180 genes that contain DTUs in two datasets, 72 genes that contain a DTU in three datasets, and 30 genes that contain a DTU in all four datasets. Thus, there are 282 genes that are significantly differentially spliced in at least two out of the four datasets (**Table S2**). In accordance, the number of junctions in a DTU that are shared between one, two, three or four datasets is 2902, 1104, 642, and 308, respectively (**Tables S3**–**S6**).

**Figure 1:**
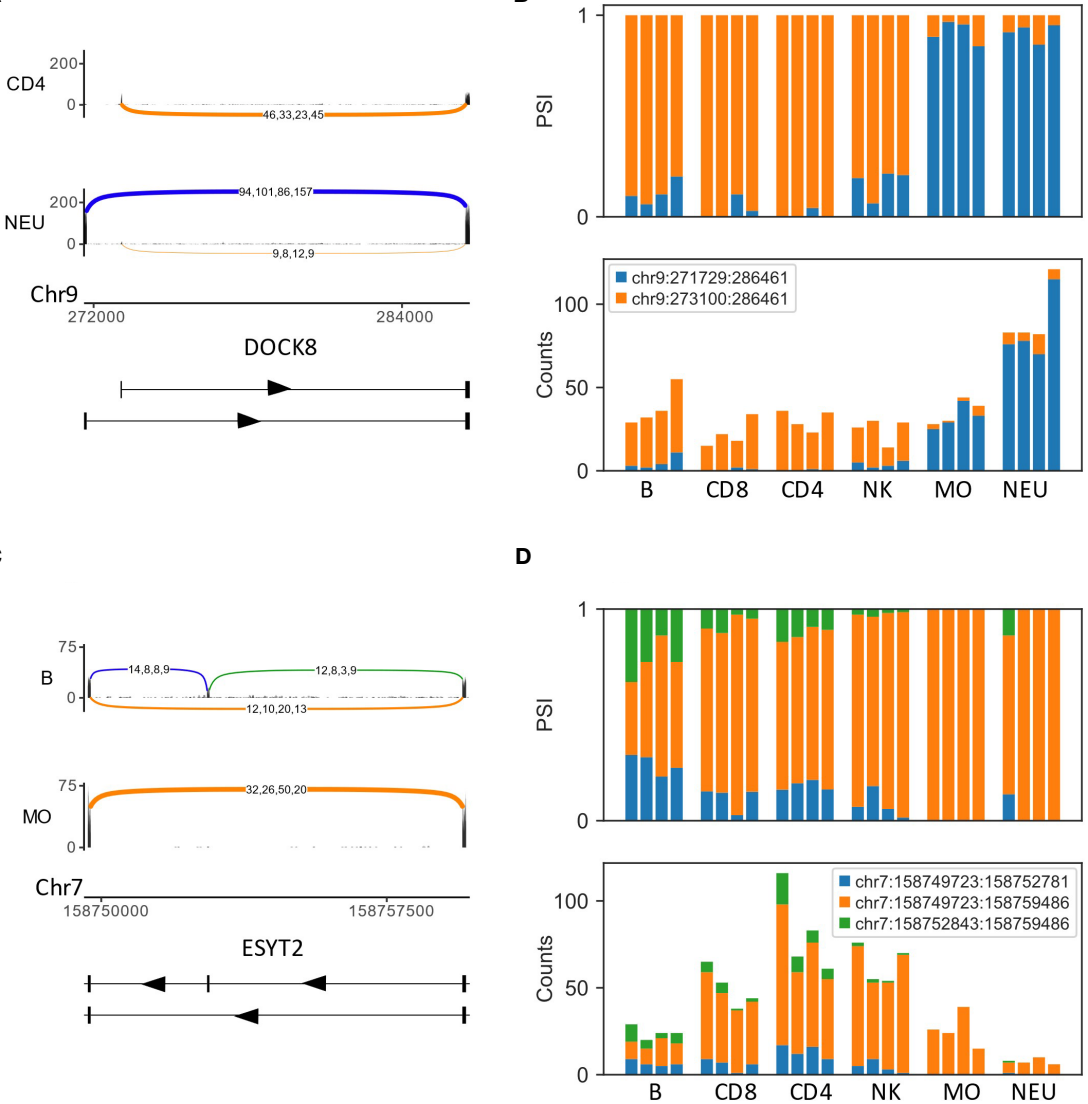
Examples of differentially spliced genes in the human immune system, GSE60424 dataset **(A)** An alternative first exon (AFE) in DOCK8. The Sashimi plots of CD4 T cells (top) and neutrophils (NEU; bottom) display the number of mapped reads; the splice junctions are shown as arcs. Samples from the same cell type are overlaid on one another. The numbers on each arc are the junction spanning read (JSR) counts of each sample. The order of the samples is the same for all junctions. **(B)** Bar plots of the fraction (top) and counts (bottom) of JSRs in DOCK8 AFE differentially spliced LeafCutter clusters (DS-LC) in all samples of all cell types. The JSRs of each junction are colored in the color of the arc of that junction in subfigure **(A). (C)** Sashimi plots of the skipped exon in ESYT2 [B cells and monocytes (MO)]. **(D)** Bar plots of the fraction (top) and counts (bottom) of JSRs of ESYT2 skipped exon DS-LC.

The analysis of different cell types enabled the identification of cell-type-specific and lineage-specific (in the context of the five cell types tested) use of splicing events. Several cases of AFE in which each exon was almost exclusive to the lymphoid lineage (B, T and NK cells) or the myeloid lineage (neutrophils and monocytes) were identified, for example, DOCK8, DGKZ and CARS2 (**Figures 1A, B**, **S3**). Another example of a lineage-specific (in the context of the five cell types tested) differentially spliced gene was ESYT2, which had an exon that was skipped in the myeloid lineage but not in the lymphoid lineage (**Figures 1C, D**).

In all datasets, there were more differential splicing events between the lymphocyte and myeloid immune lineages than within each lineage, and the myeloid cells, i.e., neutrophils and monocytes, had more events between them compared to the lymphocytes, i.e., B, T, and NK cells (**Table 3**). Thus, splicing patterns reflect the hematopoietic lineage structure.

**Table 3:** Number of differential splicing events between healthy immune cell types.

Comparison type	Cell types	GSE64655	GSE60424	GSE107011	GSE115736
Within lymphocytes	B T	28	84	32	69
Within lymphocytes	B NK	44	69	37	75
Within lymphocytes	T NK	37	88	61	93
Between lineages	B Monocytes	88	157	73	111
Between lineages	T Monocytes	78	164	83	132
Between lineages	NK Monocytes	83	124	69	103
Between lineages	B Neutrophils	86	159	70	95
Between lineages	T Neutrophils	78	166	72	106
Between lineages	NK Neutrophils	85	133	60	100
Within myeloids	Monocytes Neutrophils	72	107	55	72

The DTUs also included known differential splicing cases, such as differential splicing of PTPRC between B and T cells (36) and the AFE of CIITA between monocytes and B cells (37). For the PTPRC gene, we found that it had a very complex differential splicing pattern, as its LC included eight junctions (**Figure S4**), and that different cell types expressed different transcripts of the gene.

### Experimental validation of differential splicing

As differential splicing events in the human immune system are understudied, and we present a map of differential splicing between five immune cell types based solely on RNA-seq, we sought to validate findings by an independent method. We focused on two genes, FYB1 and CD47, in which the DTUs were within the coding region and thus likely to affect the protein, have reasonable expression level, and are well studied in immune cells.

**Figure 2:**
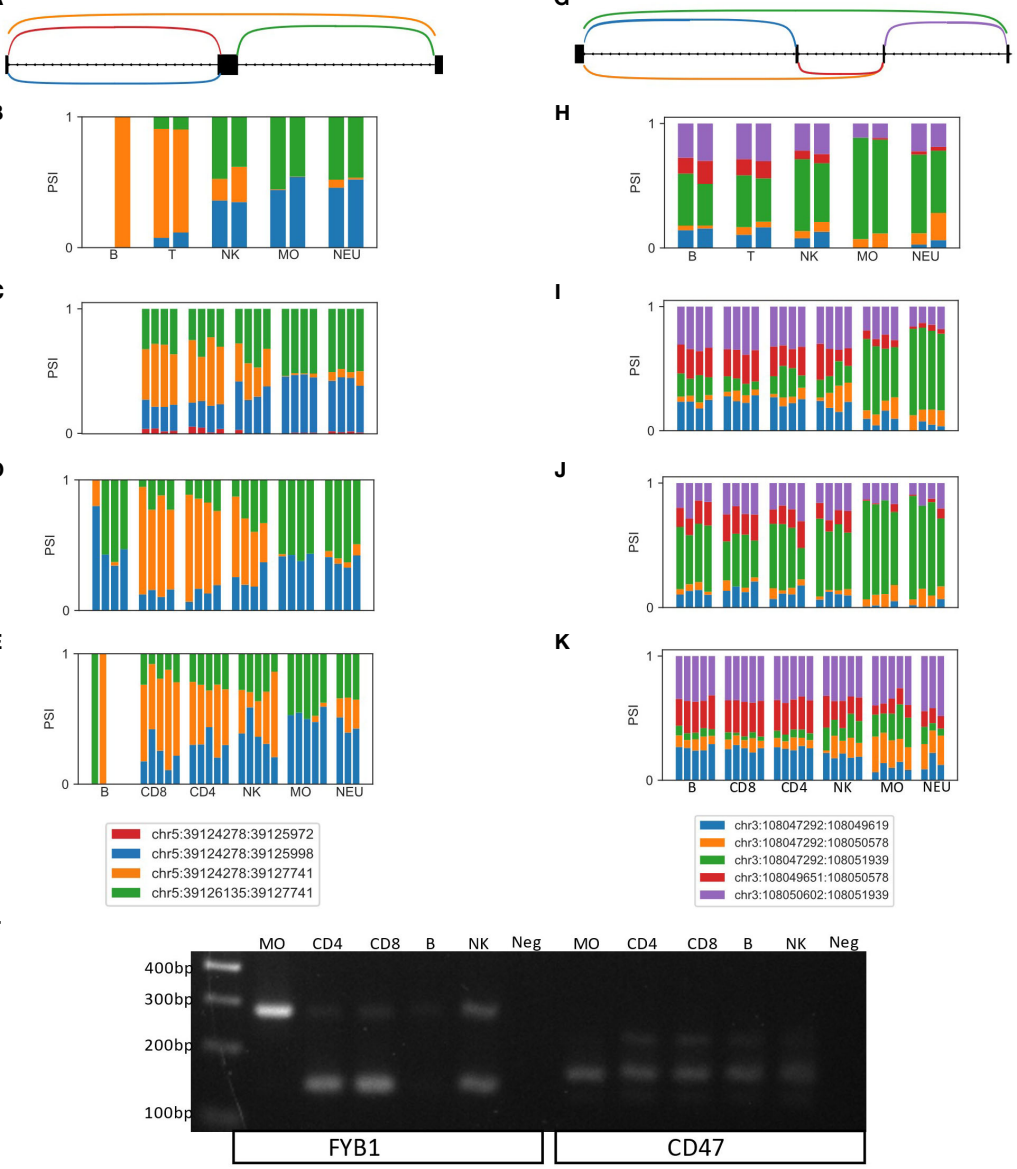
Experimentally validated examples of novel differentially spliced genes in the human immune system. **(A)** LeafCutter cluster representing a skipped exon in FYB1. **(B–E)** Bar plots of the fraction of JSRs in all samples of all cell types in datasets **(B)** GSE64655, **(C)** GSE60424 **(D)** GSE107011 and **(E)** GSE115736 are shown. The JSRs of each junction are colored in the color of the arc of that junction in **(A)**. **(F)** PCR of an independent human sample **(G)** LeafCutter cluster representing a complex alternative splicing event containing two potentially skipped exons in CD47. **(H–K)** Bar plots of the fraction of JSRs in all samples of all cell types in datasets **(H)** GSE64655, **(I)** GSE60424 **(J)** GSE107011 and **(K)** GSE115736 are shown. The JSRs of each junction are colored in the color of the arc of that junction in **(A, F)** PCR of an independent human sample with the primers marked in **(G)**.

Differential splicing of a skipped exon in FYB1 was identified in all four datasets (**Figures 2A–E**). FYB1 expression is low in B cells, but higher in the other cell types. In T cells and NKs, ~50% of the reads skip the exon (orange arc and bars in **Figures 2A–E**). However, in monocytes and neutrophiles, most reads go through the exons (blue and green arcs and bars in **Figures 2A–E**). Using primers that span that region, in T cells most of the product is the lighter one which skips the exon (145bp), whereas in NKs the levels of the two products are similar, and in monocytes the heavier product which includes the exon (283bp) is the more highly expressed, thus fully confirming the RNA-seq based alternative splicing events ratio estimations (**Figure 2F** left).

The second gene, CD47, contains a complex alternative splicing event, in which two exons can be skipped (green arc, **Figure 2G**), or one or two of those exons can be included in the transcript. In two datasets, this alternative splicing event is differentially spliced between the lineages. However, it can be seen in all four datasets that the frequency of the green arc, which skips two exons, is higher in the myeloid lineage (monocytes and neutrophiles), and the frequency of the red arc, indicating that both exons are included, is higher in the lymphocytes lineage (B, T and NK, **Figures 2H–K**). The PCR products of the two primers spanning this region indeed show only the light product (skipping both exons, 162bp) in monocytes, where lymphocytes have both the light product and the heavy product (both exons are included, 220bp, **Figure 2F** right).

### Splicing patterns in the human immune system

The identification of an LC as differentially spliced in two datasets does not necessarily imply that the splicing difference of the LC is between the same two cell types and in the same direction in the two datasets. However, for 84-94% of DS-LCs that were identified in more than one dataset, significant differences in splicing were identified in at least one shared pair of cell types in the same direction (**Table 4**, row ‘Junctions with shared comparisons’).

**Table 4:** Comparison of differential splicing between datasets.

Datasets	GSE64655 GSE60424	GSE64655 GSE107011	GSE64655 GSE115736	GSE60424 GSE107011	GSE60424 GSE115736	GSE107011 GSE115736
Shared DS junctions	233	111	121	178	244	96
Correlation of max(ΔPSI)	0.76	0.69	0.69	0.73	0.65	0.51
Junctions with shared comparisons (% of junctions)	89	84	84	87	94	90
Non-significant comparisons (% of comparisons)	35	36	35	34	29	29
Significant in one dataset (% of comparisons)	33	33	37	35	37	38
Significant in both datasets in the same direction (% of comparisons)	27	27	24	28	30	28
Significant in both datasets in opposite directions (% of comparisons)	6	4	5	3	4	5
Shared splicing factors	298	283	298	305	325	316
Shared splicing factors with correlation > 0.7 (%)	90	66	64	62	64	80
Correlation of SF-PSI	0.83	0.59	0.52	0.55	0.52	0.71
Shared transcription factors	640	591	562	563	572	507
Shared transcription factors with correlation > 0.7 (%)	95	84	74	78	77	86
Correlation of TF-PSI	0.91	0.82	0.71	0.78	0.75	0.81

**Figure 3:**
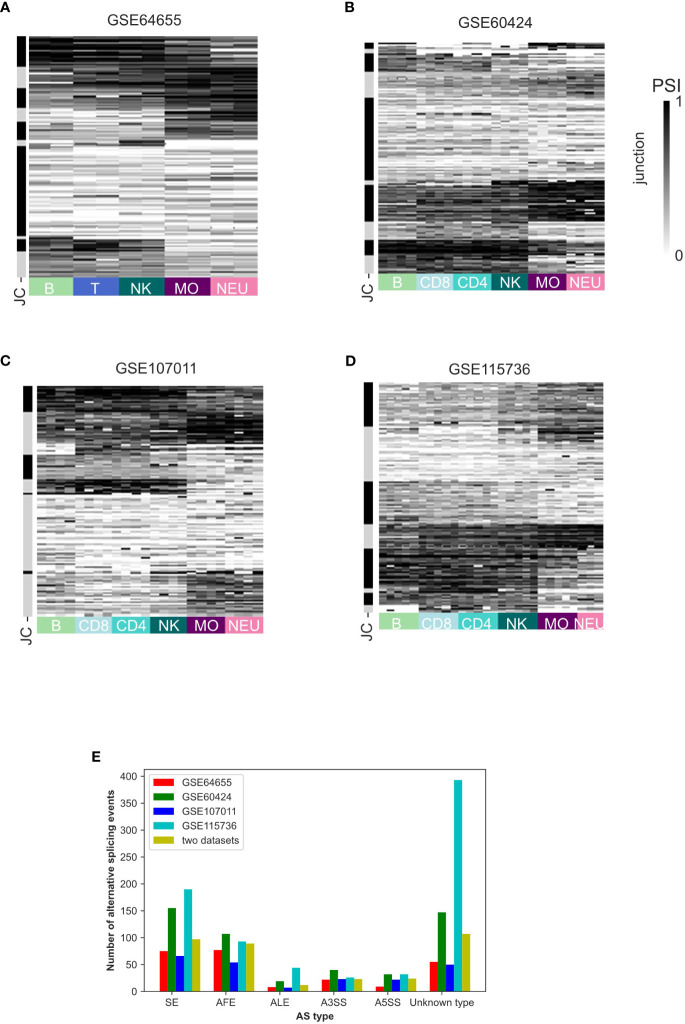
Splicing patterns in the human immune system. **(A–D)** Heatmap of the percent spliced in (PSI values) of the junctions with ΔPSI>=0.2 in differentially spliced LeafCutter clusters (DS-LC) that were identified in at least two human datasets. Each row represents a junction. Junctions in each dataset are sorted according to the clustering of the splicing patterns in that dataset. **(A)** GSE64655, **(B)** GSE60424, **(C)** GSE107011 and **(D)** GSE115736. **(E)** Bar plot of the counts of each alternative splicing (AS) type identified as differentially spliced in each dataset and in more than one dataset. DS-LCs that were not assigned alternative splicing type are labeled as an unknown type.

For each dataset, clustering of the PSI values of junctions with max(ΔPSI)>=0.2 was done separately (**Figures 3A–D**). The order of the clusters is different as they were done independently, but similar PSI patterns can be seen in all datasets. As PSI values are very noisy if the number of reads is small, we did not directly compare the PSI patterns of the shared junctions, as was done for the regulatory factors below. Instead, we compared the results of all ten comparisons between the five cell types for each of the shared junctions. About third of the comparisons are not significant in both datasets (29-36%, **Table 4**, row ‘Non significant comparisons’). About third of the comparisons are significant in only one of the datasets (33-38%, **Table 4** row ‘Significant in one dataset’), which is not surprising considering our strict filtering criteria. The last third of comparisons are significant in both datasets in the same direction (24-30%) or in different directions (3-6%). As differential splicing in different directions is most likely due to noise, it is reassuring that most findings that are identified in two datasets are in the same direction. As false positive findings are as likely to be in different directions as they are to be in the same direction, this suggests that most of the findings that are found in two datasets in the same direction are true. Additionally, the magnitude of change in splicing was also similar between the four datasets, as was evident from the high correlation of the max(ΔPSI) values of the junctions that are shared between each two datasets [0.51-0.76, **Table 4**, Correlation of max(ΔPSI) row].

The transcriptome annotation was used to assign the alternative splicing type for each of the DS-LCs in each dataset separately (**Figure 3E**; **Tables S7**). Some DS-LCs were assigned to more than one type of alternative splicing. Of note, cases of alternative splicing that involve exons or junctions that are not in the transcriptome may result in an inaccurate assignment of alternative splicing type or no assignment of alternative splicing type at all. For example, no alternative splicing type was assigned to the LC of CARS2 that is found in three datasets, as one of its junctions (chr13:110663518-110665528) is not in the NCBI annotation file used for the differential splicing analysis, although this junction is present in the Ensmbl annotation file used for **Figure S3**.

### Inference of splicing regulation in healthy cells

**Figure 4:**
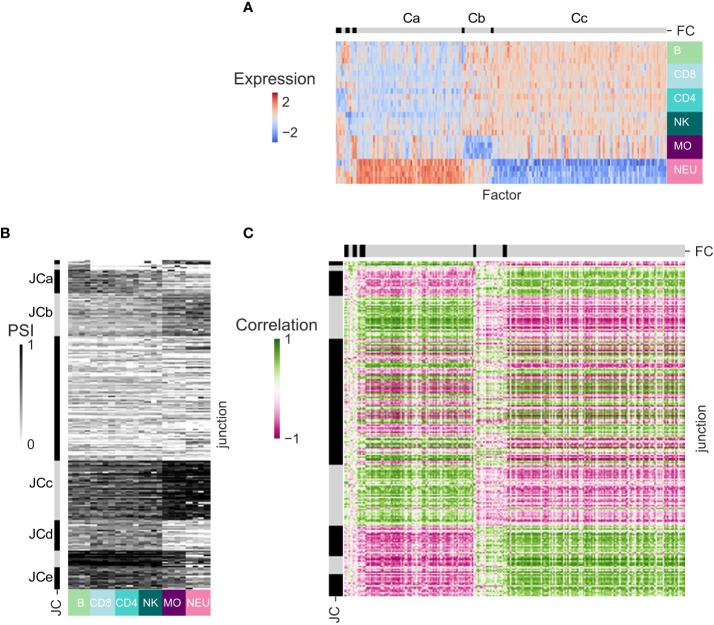
Splicing regulation inference for dataset GSE60424. **(A)** Heatmap of the expression patterns of the splicing factors (SFs) that are expressed in this dataset and at least one other dataset and are differentially expressed between immune cell types from this dataset. SFs (columns) are ordered by clustering. Clusters separation is shown on the bar at the top, and clusters discussed in text are marked by letters. **(B)** Heatmap of the splicing patterns [percent spliced in (PSI)] of the junctions that have abs(ΔPSI) ≥ 0.2 in this dataset, are identified in at least one other dataset, and are assigned an alternative splicing type that is not an alternative first exon (AFE). Junctions (rows) are ordered by clustering. Cluster separation is shown on the bar at the left, and clusters discussed in text are marked by letters. **(C)** Heatmap of the SF-junction Pearson correlation coefficients matrix.

Alternative splicing events (other than AFE) are regulated by SFs (38). We sought to associate the patterns of splicing observed across the human immune cell types with the expression patterns of known SFs. Only DS-LCs that are not classified as AFE were studied as potentially regulated by SFs. To increase robustness and for ease of interpretation, in each dataset we focused on the junctions in DS-LCs that appear in at least one more dataset and have max(ΔPSI) ≥ 0.2 in that dataset (**Table S8**). Only SFs that were expressed in at least one additional dataset and were differentially expressed between the immune cells of the dataset were considered (**Table S9**). Clustering was done on the expression patterns of the SFs. The SFs clusters of dataset GSE60424 display several expression patterns (**Figure 4A**). SF cluster Ca contains SFs that are highly expressed in neutrophils, whereas in SF cluster Cc there are SFs with the opposite expression pattern. SF cluster Cb includes SFs that are not expressed in monocytes. Clustering was done also on the splicing patterns of differentially spliced junctions in each dataset. The junction clusters of the GSE60424 dataset (JCs; **Figure 4B**) display similar patterns to the SF clusters – JCa, JCd and JCe have lower PSI values in myeloids, and JCb and JCc have higher PSI values in myeloids. Different JCs can contain differentially spliced junctions of the same gene, as they display different splicing patterns. Opposing patterns of junctions that are part of the same DS-LC are expected in simple alternative splicing events.

The SF–junction correlation matrix displayed highly correlated SF–junction blocks, corresponding to SFs that were upregulated in neutrophils and junctions that had higher PSI values in myeloid cells (Ca-JCb, Ca-JCc, **Figure 4C**) or to SFs that were downregulated in neutrophils and junctions that had higher PSI values in lymphocytes (Cc-JCa, Cc-JCd and Cc-JCe) (**Table S10**).

The patterns of SFs and junctions and the SF-junction correlation structure in the other three datasets were qualitatively similar (**Figures S5**–**S7**; **Tables S11**–**S19**). As 62-90% of the SF shared between each pair of datasets display a highly correlated pattern of expression [see row ‘Shared splicing factors with correlation > 0.7 (%)’, **Table 4**], and 84-94% of the junctions are differentially spliced in at least one comparison between the same cell types in the same direction in each dataset pairs (see row ‘Junctions with shared comparisons’, **Table 4**), it is not surprising that the Pearson correlation coefficients between the SF–PSI correlation coefficients of each pair of datasets are in the range of 0.52-0.83 (see row ‘Correlation of SF-PSI’, **Table 4**), indicating that the SF-PSI correlation structure is robust across the four datasets. Both positive and negative correlation coefficients between junction PSI values and SF expression patterns suggest that a splicing junction is potentially regulated by a SF.

An example of the predictions proposed by the SF–differentially spliced gene correlation matrix is the skipped exon of the ESYT2 gene. The junction that skips an exon in ESYT2 (chr7:158749723:158759486) is regulated by ESRP2, QKI and RBFOX1 (39–41). ESRP2 and RBFOX1 are not expressed in the GSE604242 dataset. Thus, QKI is the only known regulator of ESYT2 splicing that is expressed in GSE60424 dataset, and the splicing pattern of the ESYT2 skipped exon junction was positively correlated with QKI expression (correlation = 0.55, **Table S10**). QKI expression was also correlated with the differential slicing pattern of the two junctions in QKI gene itself (correlation = 0.63 and -0.63, **Table S10**).

### Inference of transcriptional regulation of alternative promoters in healthy immune cells

**Figure 5:**
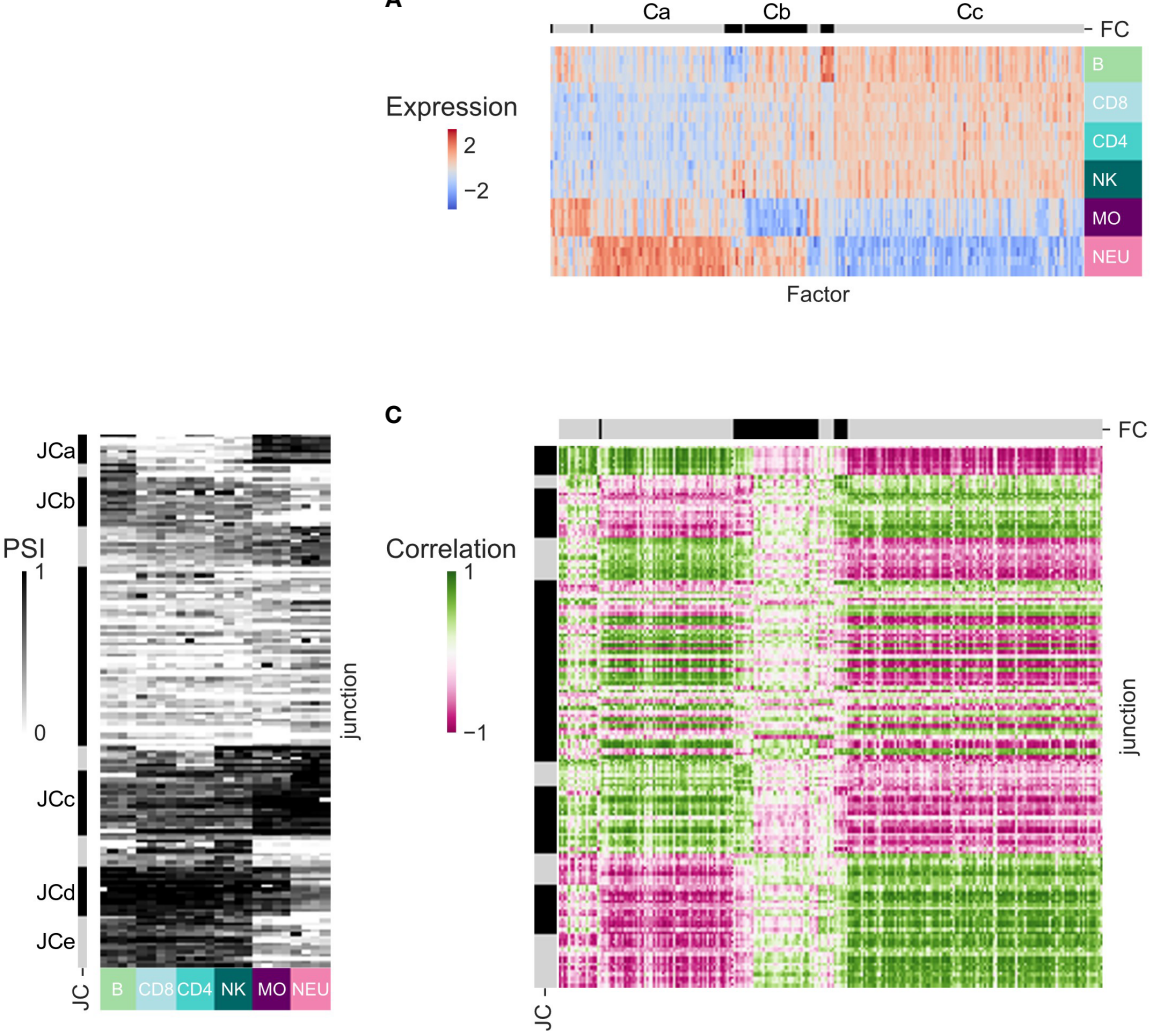
Transcription regulation inference for dataset GSE60424. **(A)** Heatmap of the expression patterns of the transcription factors (TFs) that are expressed in this dataset and at least one other dataset and are differentially expressed between immune cell types from this dataset. TFs (columns) are ordered by clustering. Cluster separation is shown on the bar at the top, and clusters discussed in text are marked by letters. **(B)** Heatmap of the splicing patterns (PSI) of the junctions that have abs(ΔPSI) ≥ 0.2 in this dataset, are identified in at least one other dataset, and were annotated as AFE. Junctions (rows) are ordered by clustering. Clusters names are shown on the bar at the left. **(C)** Heatmap of the TFs-junction Pearson correlation matrix.

The correlation between the expression patterns of TFs and the expression patterns of genes is often used for inference of potential transcriptional regulation (42). As the use of AFEs is regulated by TFs and not SFs, we sought to associate the patterns of PSI of AFEs across human immune cell types with the expression pattern of known TFs. For each dataset, we focused on the junctions in DS-LCs shared with at least one more dataset that are AFE junctions and have max(ΔPSI) ≥ 0.2 and the TFs that are expressed in at least one more dataset and are differentially expressed between different immune cell types (**Figures 5A, B**; **S8**–**S10**, **Tables S20**–**S31**). In GSE60424, TF cluster Ca included TFs that were highly expressed in neutrophils. TF cluster Cb included TFs that were downregulated in monocytes. Cc contained TFs that are upregulated in lymphocytes. Clustering the PSI patterns of the AFE differentially spliced junctions identified interesting clusters of junctions (JC). JCa and JCc included junctions that had lower PSI values in lymphocytes. JCb and JCe included junctions that had lower PSI values in myeloid cells. The correlation matrix between the expression patterns of TFs in all samples and the PSI patterns of the DS junctions that were annotated as AFEs also displays a block structure, corresponding to modules of TF and junctions with similar patterns across cell types (**Figure 5C**). In the same DS-LCs, different junctions can display different, sometimes opposing, splicing patterns (**Figure 5C**).

The correlation structure of AFE PSI patterns and TF expression patterns is robust in all four datasets. Of the TFs shared between each pair of datasets, 74-95% display a highly correlated pattern of expression (see row ‘Shared transcription factors with correlation > 0.7’, **Table 4**), and the Pearson correlation coefficients between the TF–PSI correlation coefficients of each pair of datasets are in the range of 0.71-0.91 (see row ‘Correlation of TF-PSI’, **Table 4**). Similarly to the splicing regulation above, both positive and negative correlation coefficients between junction PSI values and TF expression patterns may suggest that a promotor is potentially regulated by a TF.

### Differential splicing analysis in immune-related conditions

**Figure 6:**
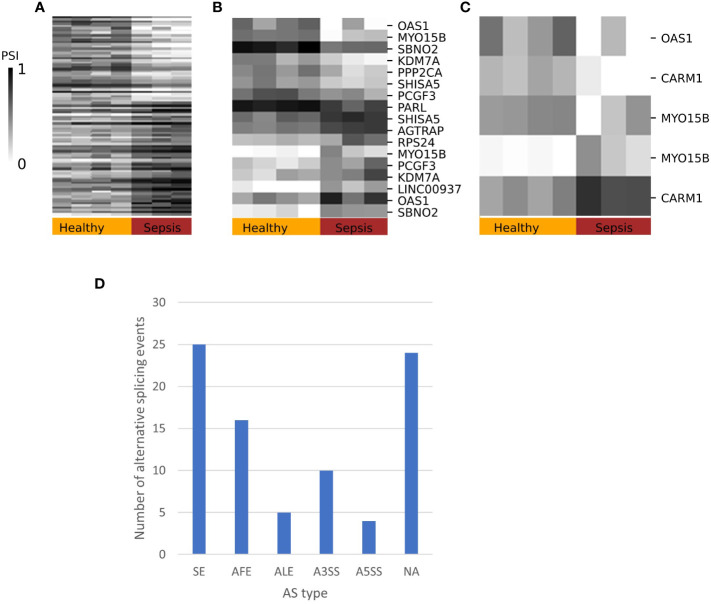
Splicing patterns in sepsis. Heatmaps of the percent spliced in (PSI values) patterns of differentially spliced junctions with ΔPSI ≥ 0.2 in sepsis compared to healthy immune cells in **(A)** neutrophils, **(B)** monocytes, and **(C)** B cells. Selected DS are annotated with gene symbol. **(D)** Distribution of differentially spliced LeafCutter clusters (DS-LCs) into alternative splicing (AS) types. DS-LCs that were not assigned alternative splicing type are labeled as an unknown type.

**Figure 7:**
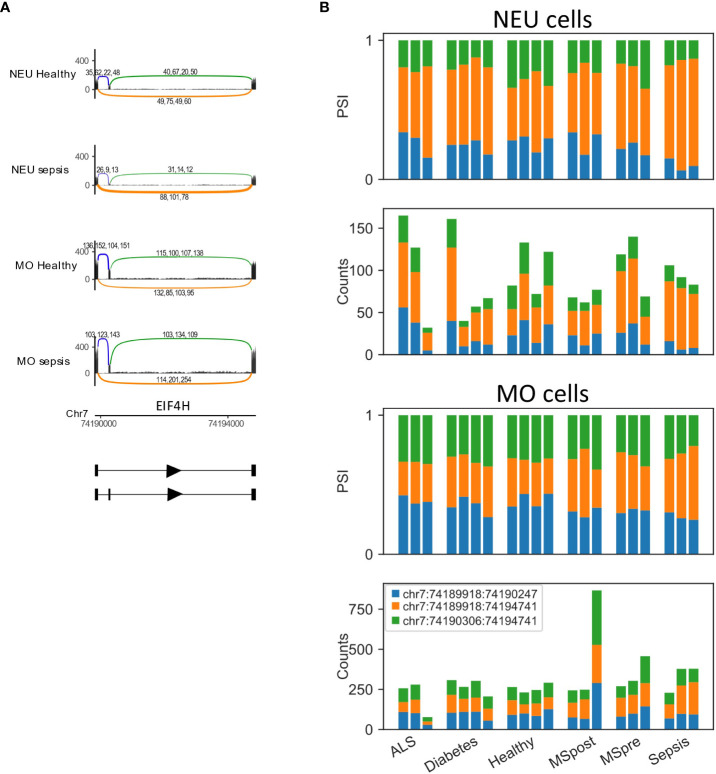
EIF4H is differentially spliced in sepsis. **(A)** Skipped exon in EIF4H. The Sashimi plots of neutrophils (NEU; healthy and sepsis, top) and monocytes (MO; healthy and sepsis, bottom) display the number of mapped reads; the splice junctions are shown as arcs. Samples from healthy or sepsis are overlaid on one another. The numbers on each arc are the junction spanning read (JSR) counts of each sample. The order of the samples is the same for all junctions. **(B)** Bar plots of EIF4H skipped exon differentially spliced LeafCutter cluster (DS-LC) from neutrophils (NEU; fraction and counts of JSR in all samples, top) and monocytes (MO; fraction and counts of JSR in all samples, bottom) from all samples. The JSRs of each junction are colored in the colors of the arcs of that junction in **(A)**.

**Figure 8:**
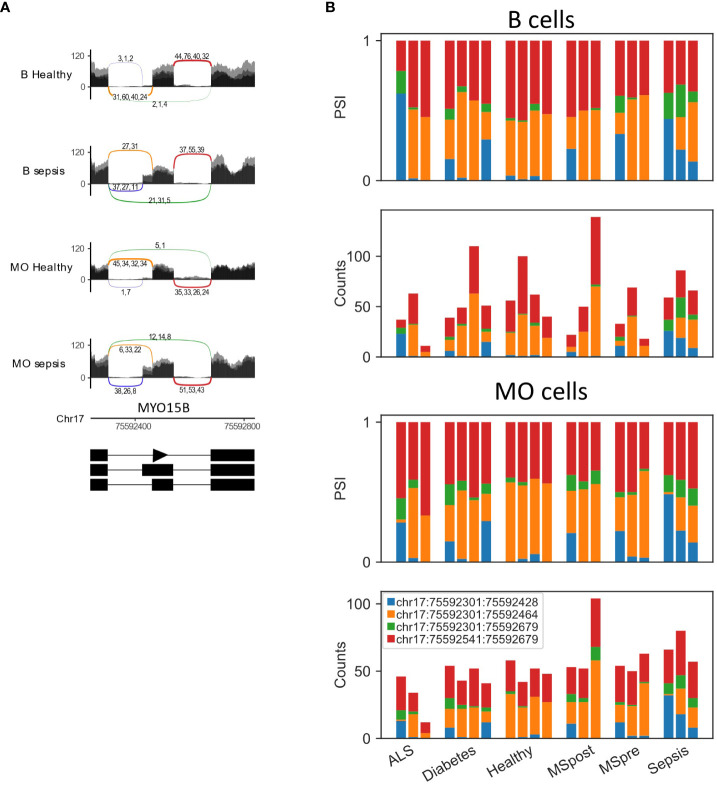
MYO15B is differentially spliced in sepsis. **(A)** Sashimi plots of the alternative 3’ splice site and skipped exon in MYO15B in healthy and sepsis B cells and monocytes (MO). **(B)** Bar plots of the fraction and counts of junction spanning reads (JSRs) of MYO15B alternative 3’ splice site and skipped exon in B cells and MO from all the samples.

To test whether changes in splicing occur in immune-related conditions, we used the GSE60424 dataset, which includes six cell types (B, CD4, CD8, NK, neutrophils, and monocytes) from healthy donors and donors with different pathological conditions, namely, ALS, type 1 diabetes, MS or sepsis. We searched for differential splicing in each cell type between healthy donors and each of the immune-related conditions. In total, in all comparisons, we identified 74 DS-LCs that mapped to 67 genes (**Tables S32**, **S33**). Only 16/74 (22%) of those DS-LCs were AFE, compared to 45% of LCs that were AFE between healthy immune cell types. Four LCs included only novel junctions, and in 21 other LCs the type was not classified (**Table S34**). ALS, MS and diabetes had almost no effect on the splicing patterns in any of the cell types. However, sepsis affected the splicing of 67 LCs that mapped to 61 genes compared to healthy controls. Of these 67 LCs, 3, 13, and 57 were differentially spliced in B cells, monocytes, and neutrophils, respectively (**Table 5**; **Figures 6**–**8**). Six genes were differentially spliced in more than one cell type (OAS1, MYO15B, WARS1, SHISA5, AGTRAP, and EIF4H; **Table S33**). SBNO2 was also differentially spliced in more than one cell type (monocytes and neutrophils), but in each cell type the differential splicing was in different part of the gene (**Figure S11**).

**Table 5:** Differential splicing events between immune cell types in health and in immune-related conditions.

Conditions	B	CD4	CD8	NK	Monocytes	Neutrophiles
MS pre treatment	0	1	2	N.T.	3	1
MS post treatment	1	2	0	2	2	5
Diabetes	1	0	0	0	1	0
ALS	0	0	0	0	3	0
Sepsis	3	0	0	0	13	57
MS pre vs. post treatment	1	0	0	N.T.	1	3

N.T. – not tested, less than three replicates per condition.

### MS treatment affects splicing

The GSE60424 dataset includes data for MS patients before and 24 h after the first treatment with interferon-beta. Our search for differential splicing between MS pre and post treatment in each of the cell types revealed four DS-LCs (**Tables 5**; **S35**). One DS-LC was identified in B cells but was not mapped to a gene. All three genes (TNK2, RABGAP1L and WARS1) were differentially spliced in neutrophils. WARS1 was also differentially spliced in monocytes. The AS type assigned to all three genes was AFE, and RABGAP1L was also assigned to ALE (**Figure S12**).

## Discussion

The immune system is a complex system in which information is processed differently depending on cell type, time and context. In such a system, accurate regulation of transcription and splicing is particularly important, as discussed in (4, 43, 44). In the first part of this study, we performed an in-depth analysis of DTU events in blood cells from healthy human donors collected in four independent datasets. We identified 282 cases of DTU events between the healthy immune cell types in at least two datasets, including the known differentially spliced skipped exons of PTPRC and the cell type specific promoter of CIITA. Other cases of DTU events were identified in genes with known immune functions, but without a reported role of differential splicing in immune cells e.g., DOCK8. This gene plays a critical role in the survival and function of several types of immune cells (45). DOCK8 immunodeficiency syndrome is characterized by recurrent severe infections that can be life threatening (46). Mutations in exons 32 or 36 lead to the expression of different transcripts that contribute to DOCK8-related disease (47, 48). We identified a change in the use of an AFE of DOCK8 between lymphoid cells (B, T cells, and NK) and myeloid cells (monocytes and neutrophils), which has not been reported previously.

We chose two DTUs that affect the coding region of immune related genes, FYB1 and CD47, for experimental validation. FYB1 (also known as ADAP) is an adaptor signaling protein, primarily studied in T cells. FYB1 canonical transcript excludes exon 12. The inclusion of that exon adds 46 amino acids between an EVH1-binding site and a putative nuclear localization signal (49). The longer variant of FYB1 is preferentially expressed in mature T cells and was reported to better induce target genes such as IL-2 (50). CD47 (also known as IAP) gained much attention as a “don’t eat me” signal for macrophages. The skipping of exons 9 and 10 of CD47 deletes 20 amino acids of the cytoplasmic carboxyl terminus. The CD47-202 isoform, that includes exons 9 and 10, was recently reported to be upregulated in pediatric Acute Myeloid Leukemia compared to normal cells (51). The differential splicing of both genes was validated.

A complementary approach to differential splicing analysis is transcript-level differential expression. When this approach was applied to seven primary human immune cell types, it identified 55 genes whose transcripts display different expression levels between different cell types (13). There are 20 genes identified by both the differential splicing analysis here and by the transcript-level differential expression analysis. Those genes include the well-known case of PTPRC, FYB1 which we experimentally validated, NCOA4, and SH3BP2, which were identified in all four datasets, ESYT2, FGR, RPS6KA1, SEPTIN9, ST6GAL1 and SYK, which were identified in three of the four datasets. There are multiple reasons for the discrepancies between the lists, and the results of these approaches. First, splicing changes which affect a relatively short part of the transcript are less likely to be identified when comparing transcript-level expression estimates, whereas differential splicing analysis is independent of the length of the change in the transcript. This may explain, for example, why CD47, whose longest transcript is 5292bp long and is changed by 60bp was identified as differentially spliced, but not as transcript-level differential expression gene, whereas FYB1 which is changed by 139bp (out of 4789) was identified by both approaches. Second, differential splicing analysis is unable to identify transcripts whose difference does not involve alternative splicing, which is the reason GPI, whose transcripts GPI-201 and GPI-202 were identified as differentially expressed, was not identified as differentially spliced. Third, the differential splicing method used here, LeafCutter, is unable to identify changes in inclusion of retained introns. Those reasons, as well as the difference in datasets, biological noise and technical noise probably explain the discrepancies between the 55 transcript-level differential expression genes (13) and the 282 differentially spliced genes identified in at least two datasets here.

As most of the DTUs that we identified have not been reported before, their regulation is unknown. Thus, we sought to use the correlation between PSI patterns and regulator expression patterns to infer potential regulators, namely, SFs for differential splicing, and TFs for differential promoter use. We identified SFs that were correlated with the PSI pattern of the differentially spliced junctions and TFs that were correlated with the PSI pattern of junctions indicating differential promoter use (AFE). The number of potential regulators of each DTU was large, but it can be reduced by using more samples or datasets. A curated database of SF and TF targets would increase the reliability of the results. Notably, in addition to the expression of the regulator, there are multiple factors that influence differential splicing, including the rate of transcription, the presence of strong or weak splice sites, and the accessibility of the splice sites (19). Similarly, there are multiple additional factors that influence promoter choice, including DNA methylation and histone modifications, e.g., in the case of CIITA (52). Despite the above limitations, the regulatory model of alternative transcript use in the human immune system we present here, though not experimentally validated, provides testable transcript specific regulation hypotheses.

To date, the role of differential splicing between healthy immune cell types is not fully understood, and likewise knowledge on the effect of immune-related conditions on splicing is also lacking. When comparing healthy immune cells to immune-related conditions cells (ALS, diabetes, MS and sepsis), we identified 74 cases of DTU events (67 in sepsis). Although the expression level of SMPD1, the only gene previously reported to change splicing in sepsis (25), was too low to be identified as exhibiting differential splicing in the tested dataset, 61 other genes were identified as exhibiting differential splicing in sepsis, including seven genes that were differentially spliced in more than one cell type: AGTRAP, EIF4H, SHISA5, WARS1 and SBNO2 were differentially spliced in both monocytes and neutrophils, and MYO15B and OAS1 were differentially spliced in B cells and monocytes. For SBNO2, the differential splicing was in different part of the gene in monocytes and neutrophils. Most of the above genes are known to be associated with sepsis or viral infection: AGTRAP is a key gene in sepsis (53); EIF4H protein interacts with NSP9 coronavirus protein (54); and WARS1 modulates innate immune responses and is thus considered an attractive target for the treatment of sepsis (55). AGTRAP, EIF4H and SBNO2 have been reported to be differentially spliced but not in immune cells (56), and SHISA5, WARS1 and OAS1 have been reported to be differentially spliced across immune system lineages (21). Another interesting DS-LC that was found is the long non coding RNA LINC00937, which is involved in the host response to viral infection (57). As very little is known about alternative splicing in MS (58), we searched for splicing changes between immune cells from MS patients before and after treatment. We found four DS-LCs in MS between pre and post treatment, and three of those DS-LCs mapped to known genes. The three differentially spliced genes (TNK2, WARS1, RABGAP1L) are known to be related to MS (59) and are differentially spliced in neutrophils. It is also known that neutrophils play a role in the pathogenesis of MS (60). An example of how the treatment changes the promoter choice is shown for RABGAP1L in neutrophils in **Figure S12**, where RABGAP1L is one of the miR-223 targets in MS (61).

Two technical issues should be addressed regarding our analysis. First, the comparison of four independently produced RNA-seq datasets is subject to many technical confounders, including the human population sampled, the sorting markers and procedure, the sequencing protocol used and the normalization method. To minimize the effect of such confounders, we performed the analysis on each dataset independently and then compared the results. In addition, we only considered DTUs that appeared in more than one dataset, and are thus more likely to be of biological significance. Second, here we only used one method to identify differential splicing, LeafCutter (32), though there are several commonly used methods, for example rMATS (62) and MAJIQ (63). There are three reasons for that: (1) LeafCutter was easily extendable to multiple conditions comparison, crucial for our analysis; (2) LeafCutter identifies complex splicing events, and alternative first and last exons and (3) comparing between five cell types in four datasets using more than one method would make this analysis hard to interpret.

In the current study, we mapped differential splicing between five immune cell types, from four datasets, in health and immune-related conditions. In the healthy cell types analysis, we identified 245 cases of differential splicing and 89 cases of AFE that are found in at least two datasets, and suggested potential regulators for many of those cases. Our mapping adds an additional layer of complexity to the regulation of the healthy and diseased immune system and suggests that transcript diversity plays a critical role in controlling immune differentiation and response. As detecting DTUs requires a higher expression level compared to detecting differential expression, the extent of the contribution of DTUs to cell function in the immune system is probably an under-estimation.

## Data availability statement

Publicly available datasets were analyzed in this study. This data can be found here: https://www.ncbi.nlm.nih.gov/geo/query/acc.cgi?acc=GSE60424, https://www.ncbi.nlm.nih.gov/geo/query/acc.cgi?acc=GSE64655, https://www.ncbi.nlm.nih.gov/geo/query/acc.cgi?acc=GSE107011, https://www.ncbi.nlm.nih.gov/geo/query/acc.cgi?acc=GSE115736.

## Ethics statement

The studies involving humans were approved by Ben-Gurion University ethics committee. The studies were conducted in accordance with the local legislation and institutional requirements. The participants provided their written informed consent to participate in this study.

## Author contributions

HN-G performed the analysis. RK and RG performed the experiments. AR-B developed the cell comparison analysis. TS supervised the research. All authors contributed to manuscript writing.

## Supplementary material

Supplementary Figure 1 Overall analysis. **(A)** A graphical description of the workflow. Differential splicing analysis was applied to four RNA-seq datasets which profiled five human immune cell types - B, T, natural killer cells (NK), monocytes (MO) and neutrophiles (NEU). The splicing patterns of the 282 events of differential transcript use that were identified in more than one dataset were studied. Differential splicing of two genes was experimentally validated. Finally, regulatory modeling of splicing and promotor choice were suggested for differential splicing events and alternative first exon use events (AFE), respectively. **(B)** Differential splicing analysis was performed by LeafCutter, which defines LeafCutter clusters (LCs) of junction spanning reads (JSRs). The Sashimi plot displays a LC which has two junctions with less than 10 JSRs. **(C)** The two disconnected LCs redefined after the removal of the poorly covered JSRs.

Supplementary Figure 2 The different types of alternative splicing events. **(A)** Skipped exon (SE). **(B)** Mutually exclusive exons (MXE). **(C)** Alternative 5’ splice sites (A5SS). **(D)** Alternative 3’ splice sites (A3SS). **(E)** Alternative first exons (AFE). **(F)** Alternative last exons (ALE).

Supplementary Figure 3 Lineage-specific (in the context of the five cell types tested) use of splicing events. **(A)** An AFE in DGKZ. Sashimi plots of CD8 T cells (top) and monocytes (bottom) are shown. Bars represent the number of reads mapped to each genomic position, and arcs represent splice junctions. Samples from the same cell type are overlaid on one another. The numbers on each arc are the JSR count of each sample. The order of the samples is the same for all junctions. **(B)** Bar plots of the fraction (top) and counts (bottom) of JSRs in DGKZ AFE DS-LC in all healthy samples of all cell types in the GSE60424 dataset. The JSRs of each junction are colored in the color of the arc of that junction in A. **(C)** Sashimi plots of the AFE in CARS2 in B cells (top) and neutrophils (bottom). **(D)** Bar plots of the fraction (top) and counts (bottom) of JSRs in CARS2 AFE DS-LC in all healthy samples of all cell types in the GSE60424 dataset.

Supplementary Figure 4 Example of the known differentially spliced gene, PTPRC, in the human immune system. **(A)** The Sashimi plots of B, CD4 T, CD8 T and monocytes display the number of mapped reads; the splice junctions are shown as arcs. Samples from the same cell type are overlaid on one another. The numbers on each arc are the JSR counts for each sample. The order of the samples is the same for all junctions. **(B)** Bar plots of the fraction (top) and counts (bottom) of JSRs in PTPRC DS-LC in all samples of all cell types in the GSE60424 dataset. The JSRs of each junction are colored in the color of the arc of that junction in A.

Supplementary Figure 5 Splicing regulation inference in the GSE64655 dataset. **(A)** Heatmap of the expression patterns of the splicing factors (SFs) that are expressed in at least two datasets and are differentially expressed in the immune system. SFs (columns) are ordered by clustering. **(B)** Heatmap of the splicing patterns (PSI) of the junctions that have abs(ΔPSI) ≥ 0.2 and are assigned an alternative splicing type other than AFE. Junctions (rows) are ordered by clustering. **(C)** Heatmap of the SF-junction Pearson correlation coefficients matrix.

Supplementary Figure 6 Splicing regulation inference in the GSE107011 dataset. **(A)** Heatmap of the expression patterns of the SFs that are expressed in at least two datasets and are differentially expressed in the immune system. SFs (columns) are ordered by clustering. **(B)** Heatmap of the splicing patterns (PSI) of the junctions that have abs(ΔPSI) ≥ 0.2 and are assigned an alternative splicing type other than AFE. Junctions (rows) are ordered by clustering. **(C)** Heatmap of the SF-junction Pearson correlation coefficients matrix.

Supplementary Figure 7 Splicing regulation inference in the GSE115736 dataset. **(A)** Heatmap of the expression patterns of the SFs that are expressed in at least two datasets and are differentially expressed in the immune system. SFs (columns) are ordered by clustering. **(B)** Heatmap of the splicing patterns (PSI) of the junctions that have abs(ΔPSI) ≥ 0.2 and are assigned an alternative splicing type other than AFE. Junctions (rows) are ordered by clustering. **(C)** Heatmap of the SF-junction Pearson correlation coefficients matrix.

Supplementary Figure 8 Transcription regulation inference in the GSE64655 dataset. **(A)** Heatmap of the expression patterns of the transcription factors (TFs) that are expressed in at least two datasets and are differentially expressed in the immune system. TFs (columns) are ordered by clustering. **(B)** Heatmap of the splicing patterns (PSI) of the junctions that have abs(ΔPSI) ≥ 0.2 and were annotated as AFE. Junctions (rows) are ordered by clustering. **(C)** Heatmap of the TF-junction Pearson correlation coefficients matrix.

Supplementary Figure 9 Transcription regulation inference in the GSE107011 dataset. **(A)** Heatmap of the expression patterns of the TFs that are expressed in at least two datasets and are differentially expressed in the immune system. TFs (columns) are ordered by clustering. **(B)** Heatmap of the splicing patterns (PSI) of the junctions that have abs(ΔPSI) ≥ 0.2 and were annotated as AFE. Junctions (rows) are ordered by clustering. **(C)** Heatmap of the TF-junction Pearson correlation coefficients matrix.

Supplementary Figure 10 Transcription regulation inference in the GSE115736 dataset. **(A)** Heatmap of the expression patterns of the TFs that are expressed in at least two datasets and are differentially expressed in the immune system. TFs (columns) are ordered by clustering. **(B)** Heatmap of the splicing patterns (PSI) of the junctions that have abs(ΔPSI) ≥ 0.2 and were annotated as AFE. Junctions (rows) are ordered by clustering. **(C)** Heatmap of the TF-junction Pearson correlation coefficients matrix.

Supplementary Figure 11 Differential splicing of SBNO2 DS-LCs in sepsis. **(A)** Sashimi plots of control and sepsis monocytes from SBNO2 chr19:1127765-1147309. **(B)** Sashimi plots of control and sepsis neutrophils from SBNO2 chr19:1154402-1174172. Sashimi plots display the mapped reads number; the splice junctions are shown as arcs. Samples from the control or sepsis donors are overlaid on one another. The numbers on each arc are the JSR counts of each sample. The order of the samples is the same for all junctions.

Supplementary Figure 12 Differential transcript use of RABGAP1L in MS. The Sashimi plots of healthy, pre-treatment MS and post-treatment MS neutrophiles display the number of mapped reads; the splice junctions are shown as arcs. The numbers on each arc are the JSR counts of each sample. The transcript structure at the bottom displays both alternative first exon and alternative last exon.
