# Supplementary material for: Mapping the splicing landscape of the human immune system
Source: Front Immunol. 2023 Aug 30;14:1116392. doi: 10.3389/fimmu.2023.1116392 (PMC10499523; doi:10.3389/fimmu.2023.1116392)
Supplement: Supplementary Figure 1 — Overall analysis. (A) A graphical description of the workflow. Differential splicing analysis was applied to four RNA-seq datasets which profiled five human immune cell types - B, T, natural killer cells (NK), monocytes (MO) and neutrophiles (NEU). The splicing patterns of the 282 events of differential transcript use that were identified in more than one dataset were studied. Differential splicing of two genes was experimentally validated. Finally, regulatory modeling of splicing and promotor choice were suggested for differential splicing events and alternative first exon use events (AFE), respectively. (B) Differential splicing analysis was performed by LeafCutter, which defines LeafCutter clusters (LCs) of junction spanning reads (JSRs). The Sashimi plot displays a LC which has two junctions with less than 10 JSRs. (C) The two disconnected LCs redefined after the removal of the poorly covered JSRs. [file DataSheet_1.pdf]

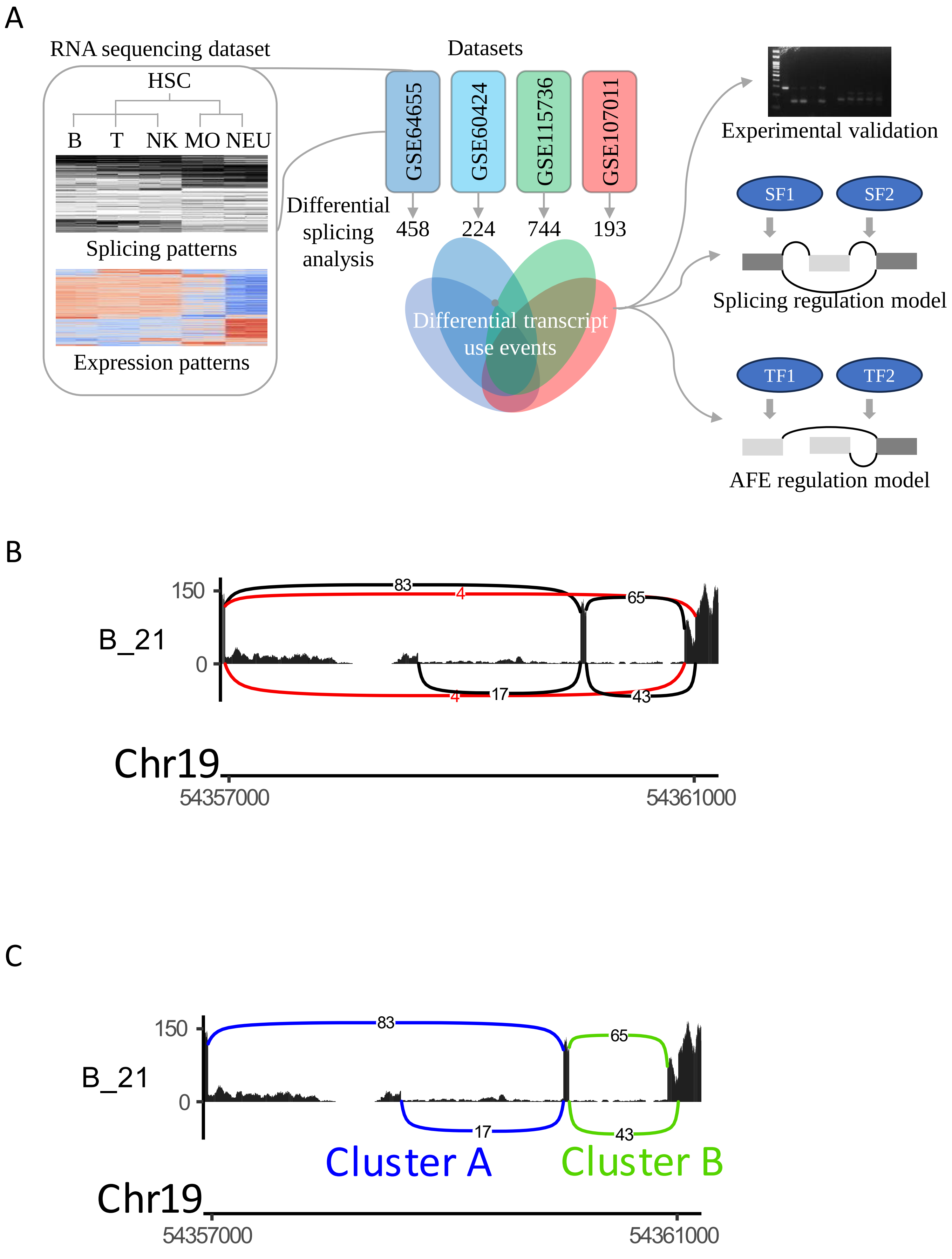

A
RNA sequencing dataset
HSC
B T NK MO NEU
Splicing patterns
Expression patterns
Datasets
GSE64655
GSE60424
GSE115736
GSE107011
Differential splicing analysis
458
224
744
193
Differential transcript use events
Experimental validation
SF1
SF2
Splicing regulation model
TF1
TF2
AFE regulation model
B
150
0
B_21
83
4
65
17
4
43
Chr19
54357000
54361000
C
150
0
B_21
83
65
17
43
Cluster A
Cluster B
Chr19
54357000
54361000

Figure S1

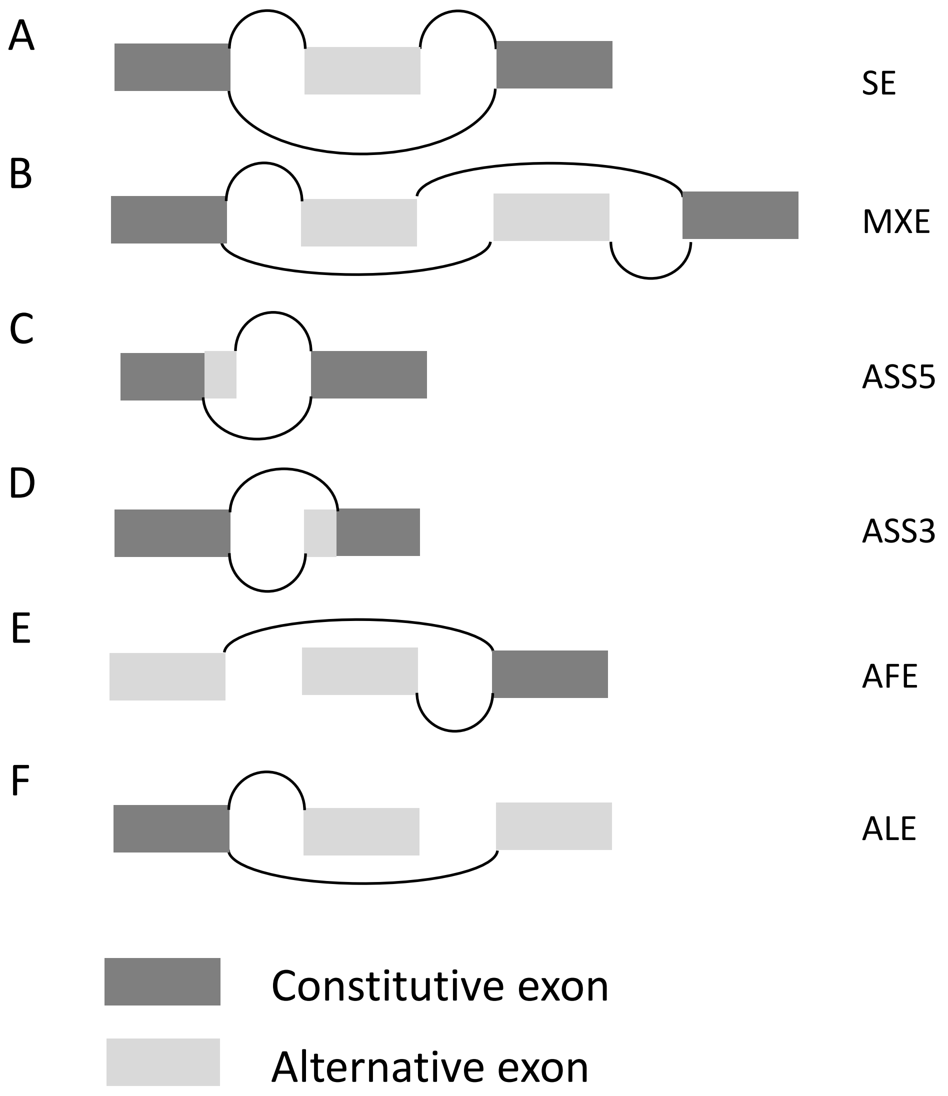
A
SE
B
MXE
C
ASS5
D
ASS3
E
AFE
F
ALE
Constitutive exon
Alternative exon

Figure S2

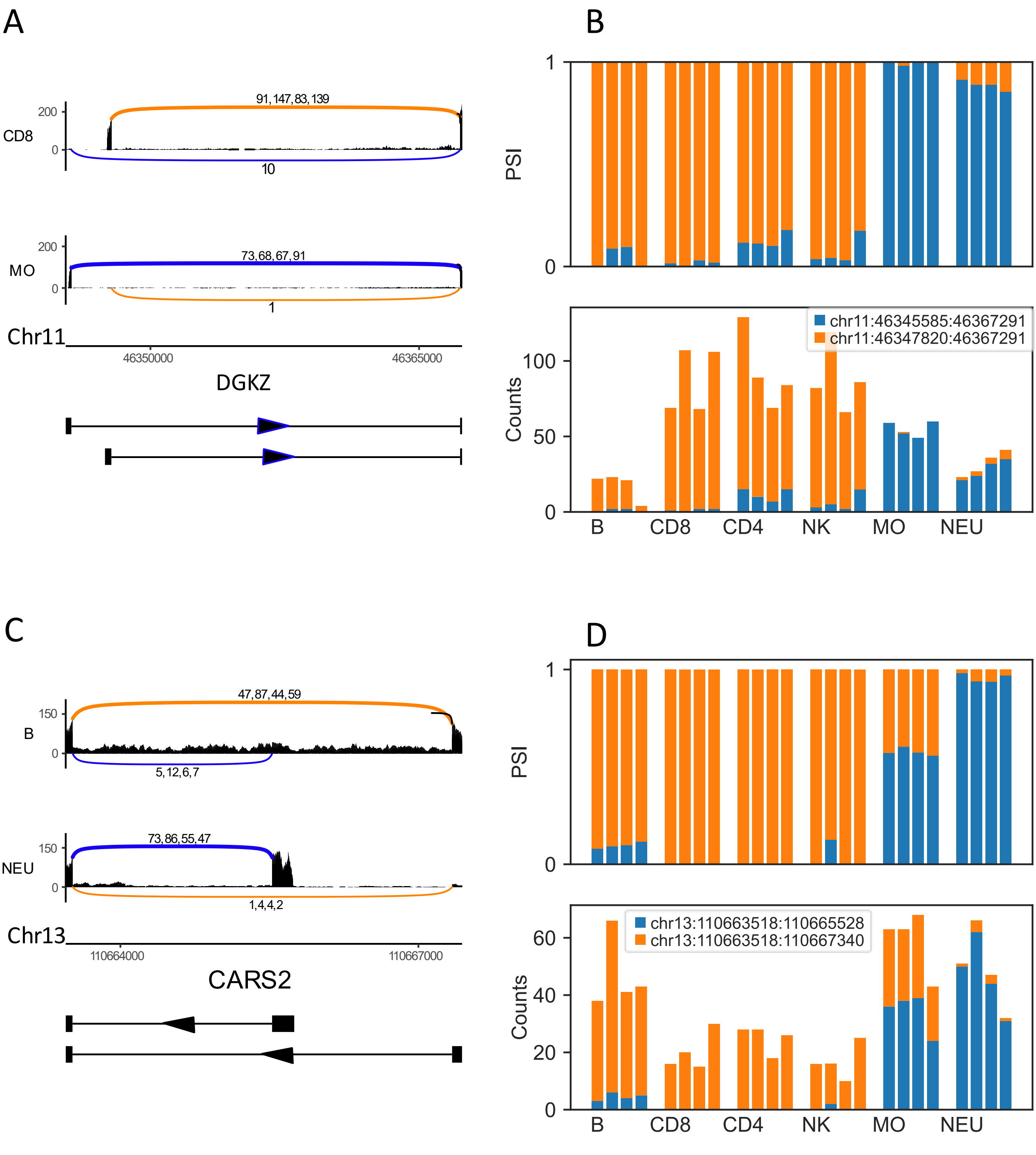
A
CD8
200
0
91, 147, 83, 139
10
MO
200
0
73, 68, 67, 91
1
Chr11
46350000
46365000
DGKZ
B
1
0
PSI
Counts
100
50
0
chr11:46345585:46367291
chr11:46347820:46367291
B
CD8
CD4
NK
MO
NEU
C
B
150
0
47,87,44,59
5,12,6,7
NEU
150
0
73,86,55,47
1,4,4,2
Chr13
110664000
110667000
CARS2
D
1
0
PSI
Counts
60
40
20
0
chr13:110663518:110665528
chr13:110663518:110667340
B
CD8
CD4
NK
MO
NEU

Figure S3

A

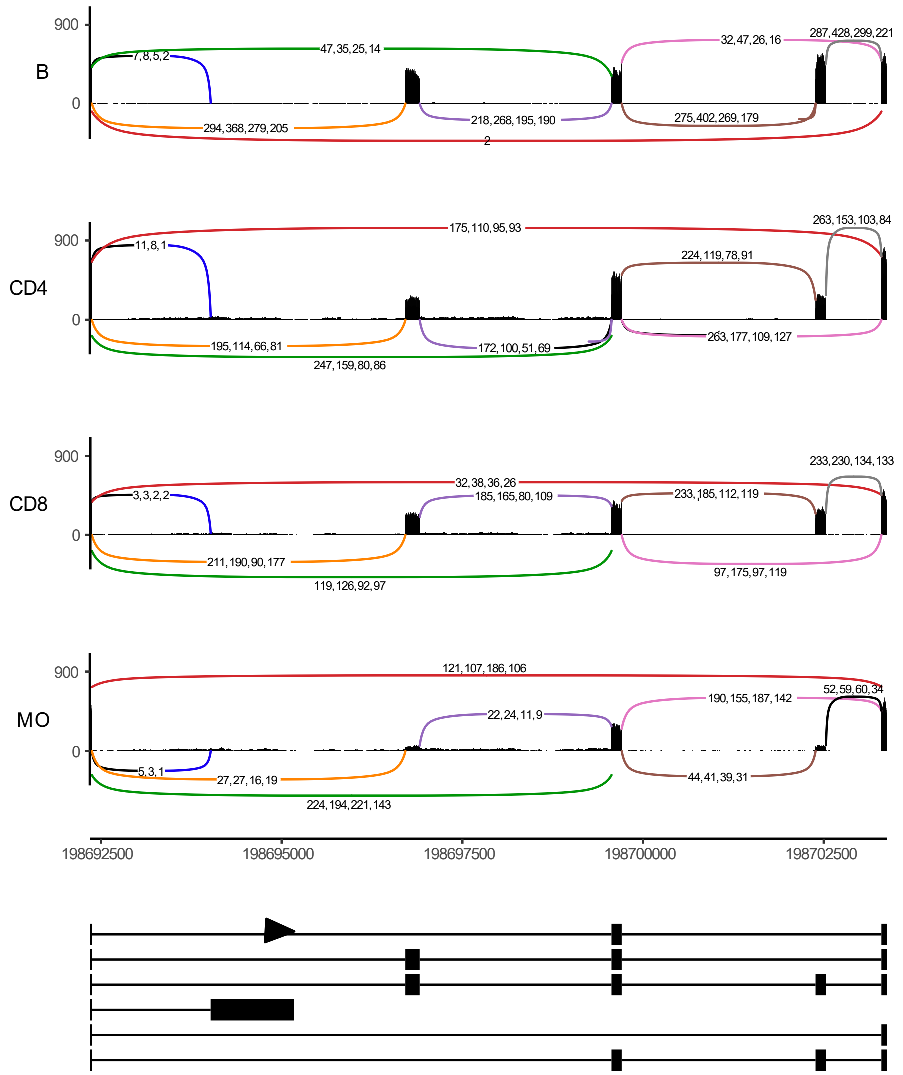

B
CD4
CD8
MO
900
0
47,35,25,14
7,8,5,2
32,47,26,16
287,428,299,221
294,368,279,205
218,268,195,190
275,402,269,179
2
175,110,95,93
11,8,1
263,153,103,84
224,119,78,91
195,114,66,81
172,100,51,69
263,177,109,127
247,159,80,86
3,3,2,2
32,38,36,26
185,165,80,109
233,185,112,119
233,230,134,133
211,190,90,177
119,126,92,97
97,175,97,119
121,107,186,106
52,59,60,34
190,155,187,142
22,24,11,9
5,3,1
27,27,16,19
44,41,39,31
224,194,221,143
198692500
198695000
198697500
198700000
198702500

B

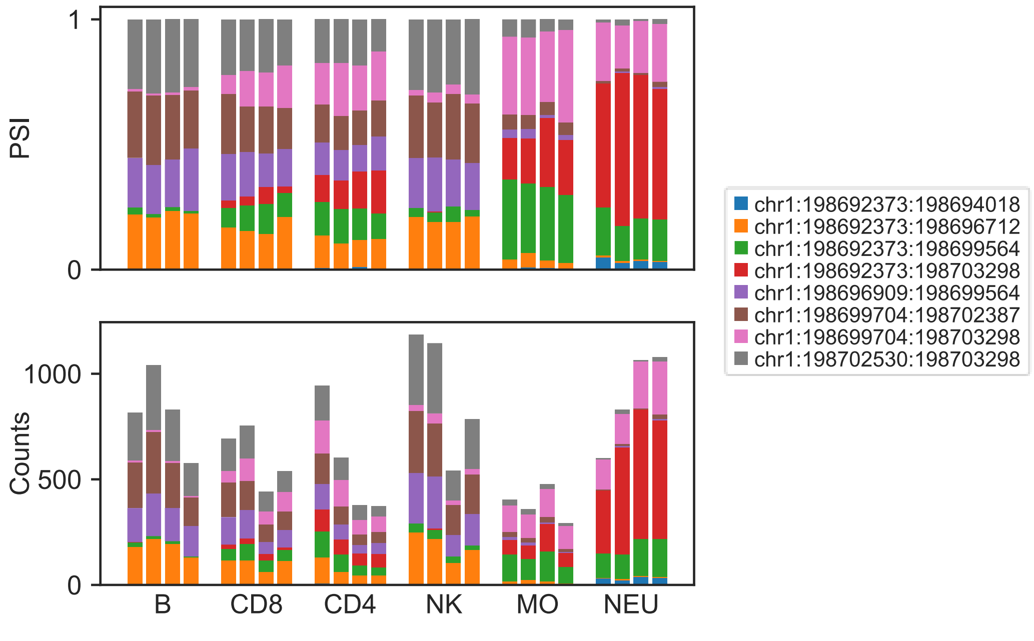

PSI
1
0
Counts
1000
500
0
B
CD8
CD4
NK
MO
NEU
chr1:198692373:198694018
chr1:198692373:198696712
chr1:198692373:198699564
chr1:198692373:198703298
chr1:198696909:198699564
chr1:198699704:198702387
chr1:198699704:198703298
chr1:198702530:198703298

Figure S4

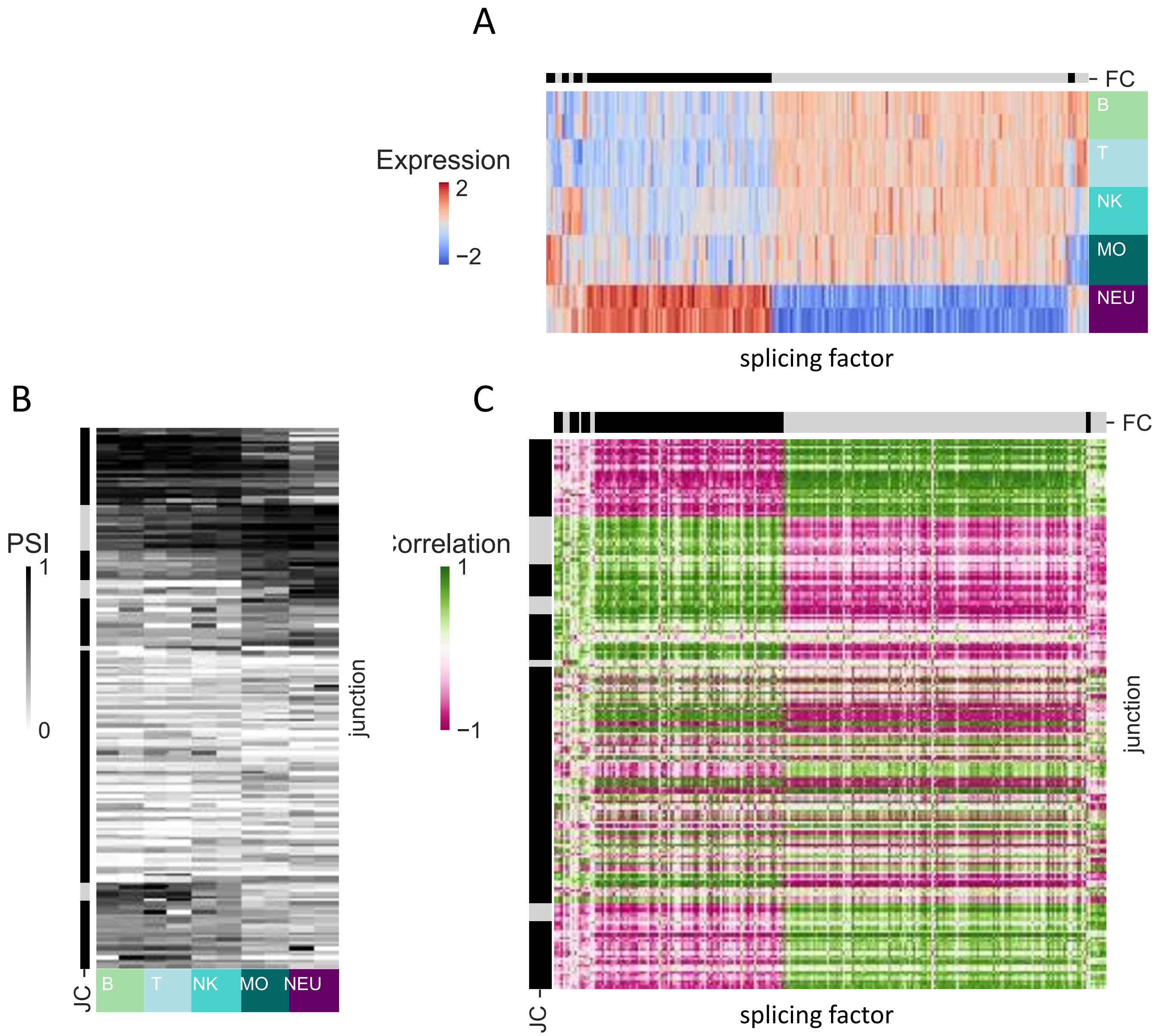
A
FC
B
T
NK
MO
NEU
Expression
2
−2
splicing factor
B
PSI
1
0
junction
JC
B
T
NK
MO
NEU
C
FC
orrelation
1
−1
junction
JC
splicing factor

Figure S5

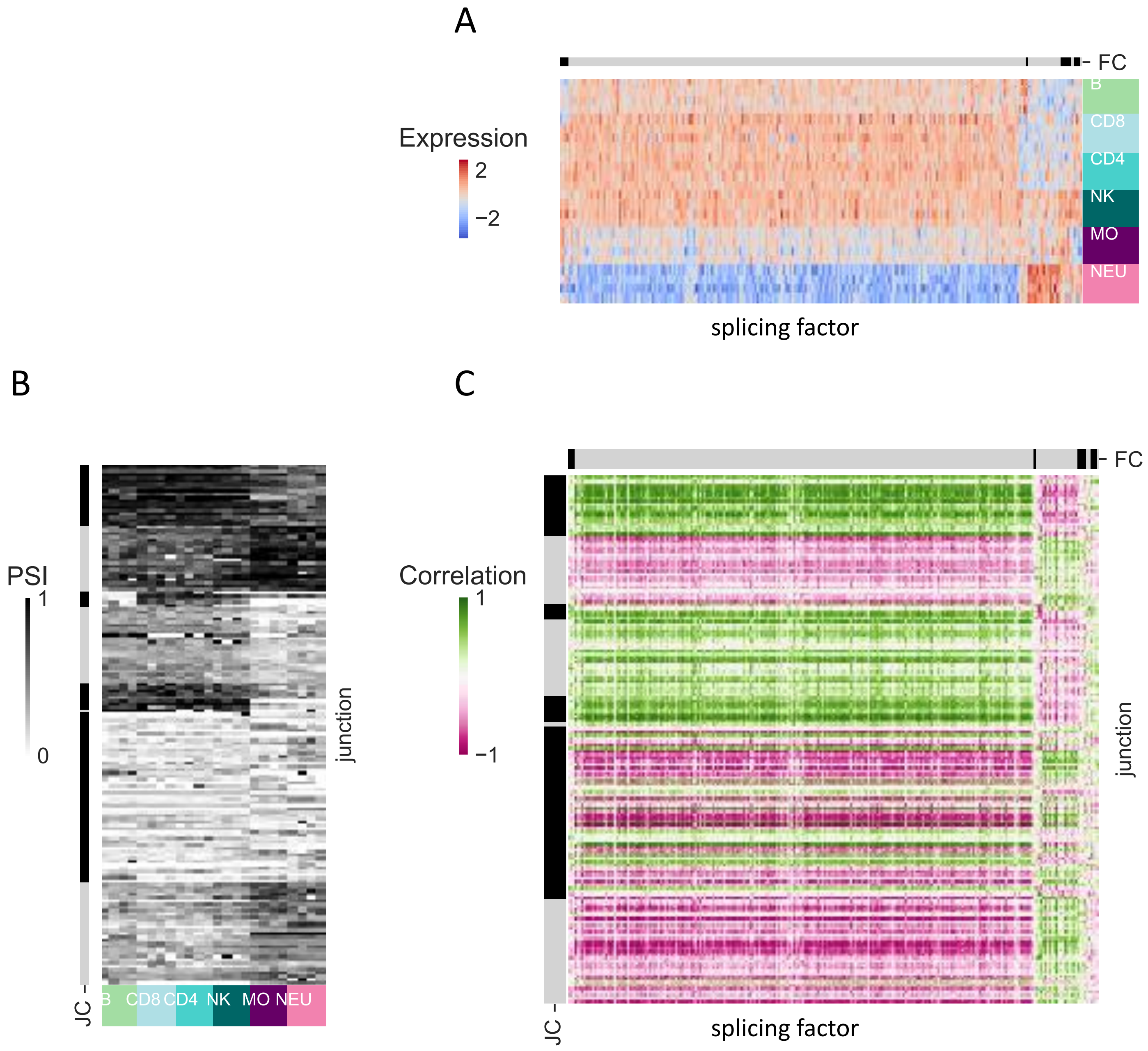
A
FC
Expression
2
−2
B
CD8
CD4
NK
MO
NEU
splicing factor
B
PSI
1
0
junction
JC
B CD8 CD4 NK MO NEU
C
FC
Correlation
1
−1
junction
JC
splicing factor

Figure S6

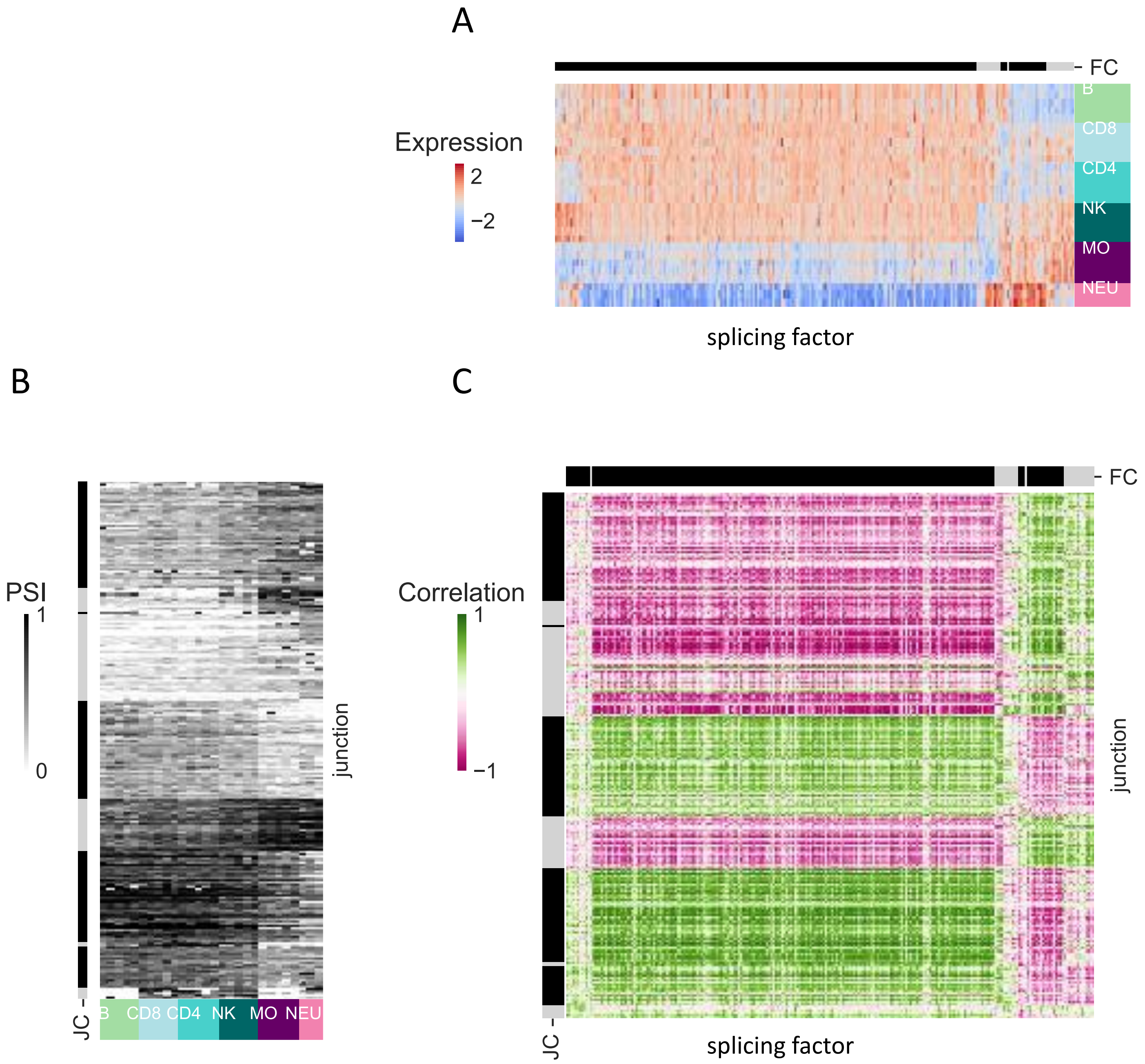
A
FC
B
CD8
CD4
NK
MO
NEU
Expression
2
−2
splicing factor
B
PSI
1
0
junction
JC
B
CD8
CD4
NK
MO
NEU
C
FC
Correlation
1
−1
junction
JC
splicing factor

Figure S7

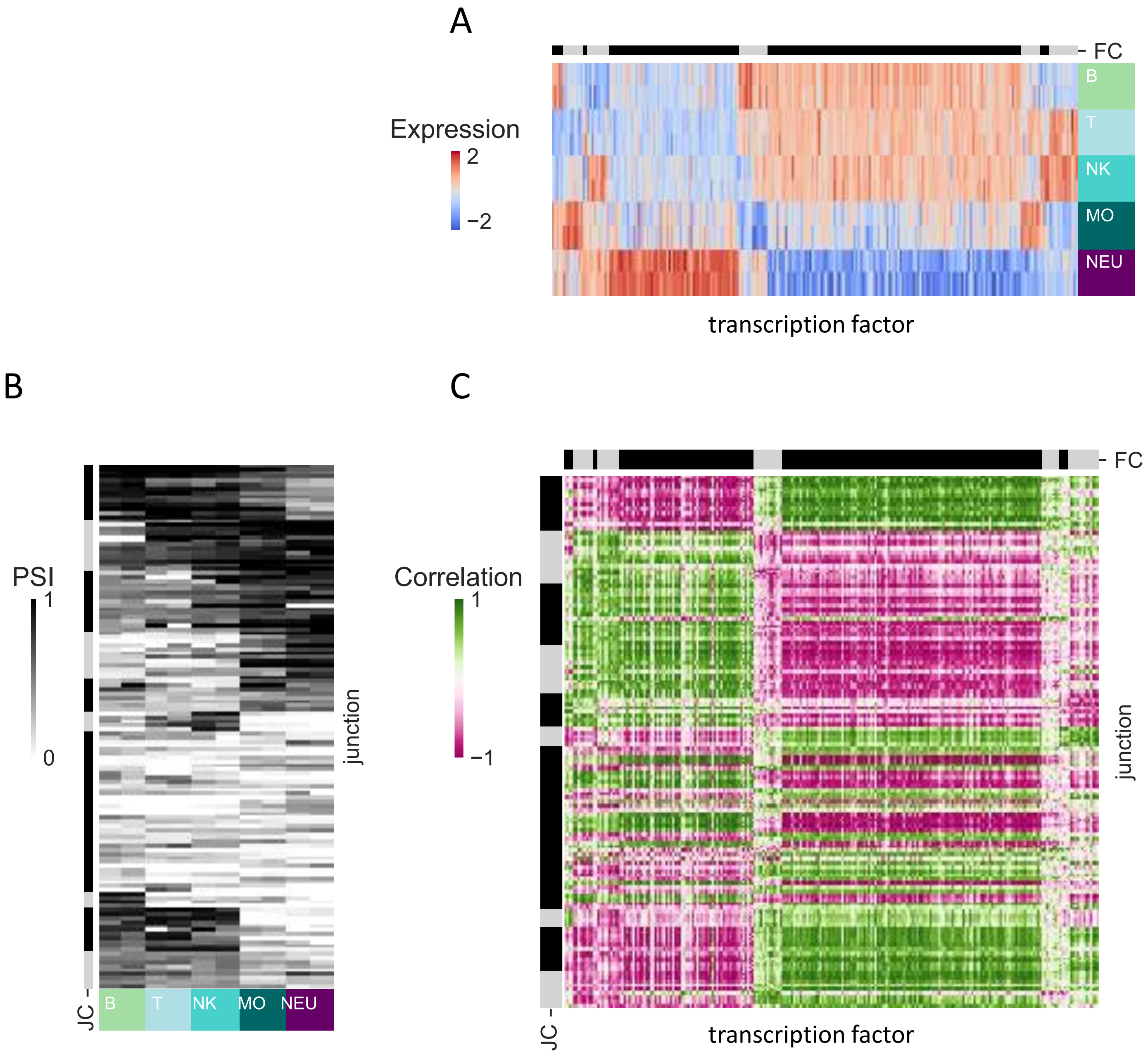
A
FC
B
T
NK
MO
NEU
Expression
2
−2
transcription factor
B
PSI
1
0
junction
JC
B
T
NK
MO
NEU
C
FC
Correlation
1
−1
junction
JC
transcription factor

Figure S8

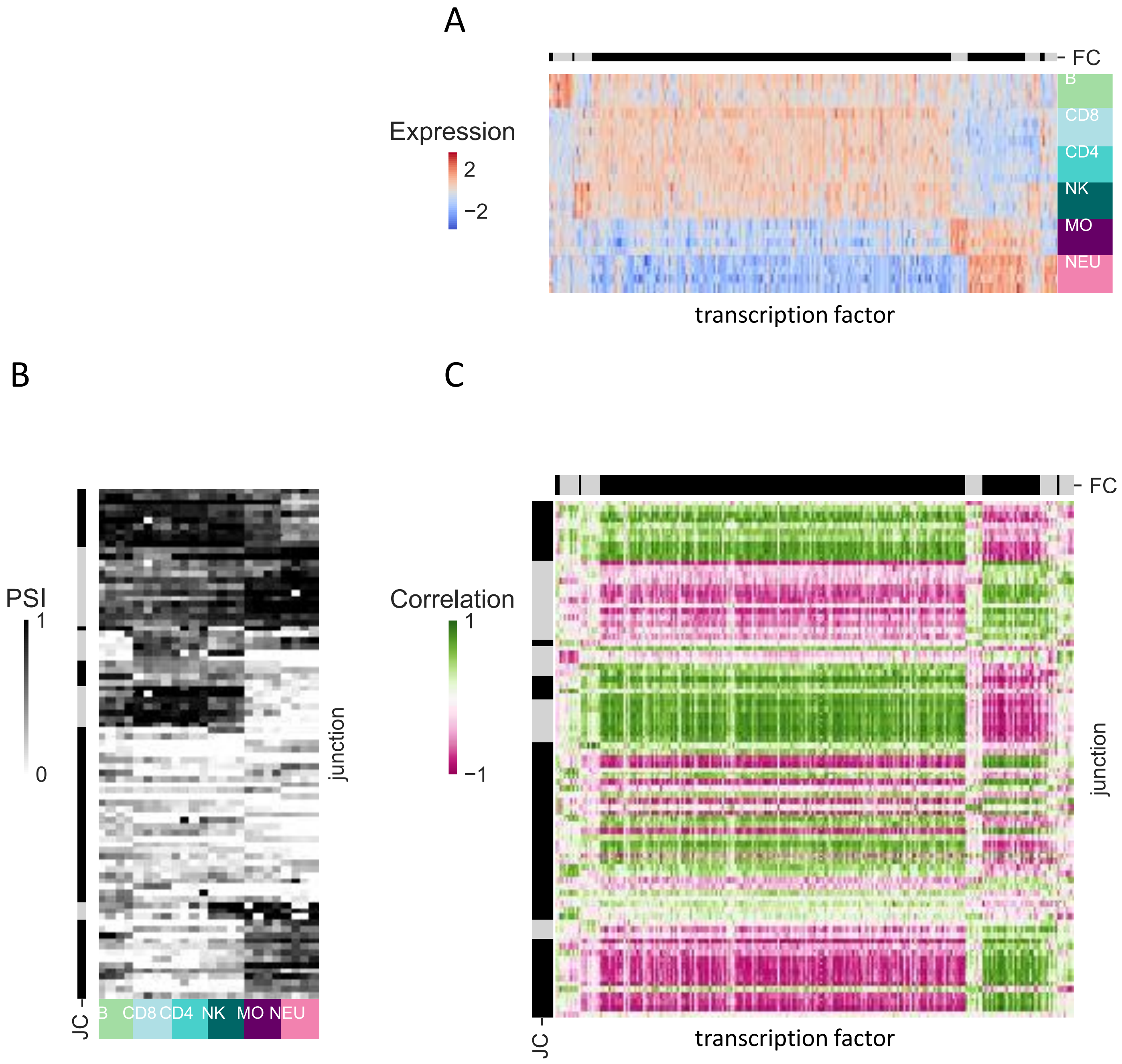

A
Expression
2
−2
FC
B
CD8
CD4
NK
MO
NEU
transcription factor
B
PSI
1
0
junction
JC
B
CD8
CD4
NK
MO
NEU
C
Correlation
1
−1
FC
junction
JC
transcription factor

Figure S9

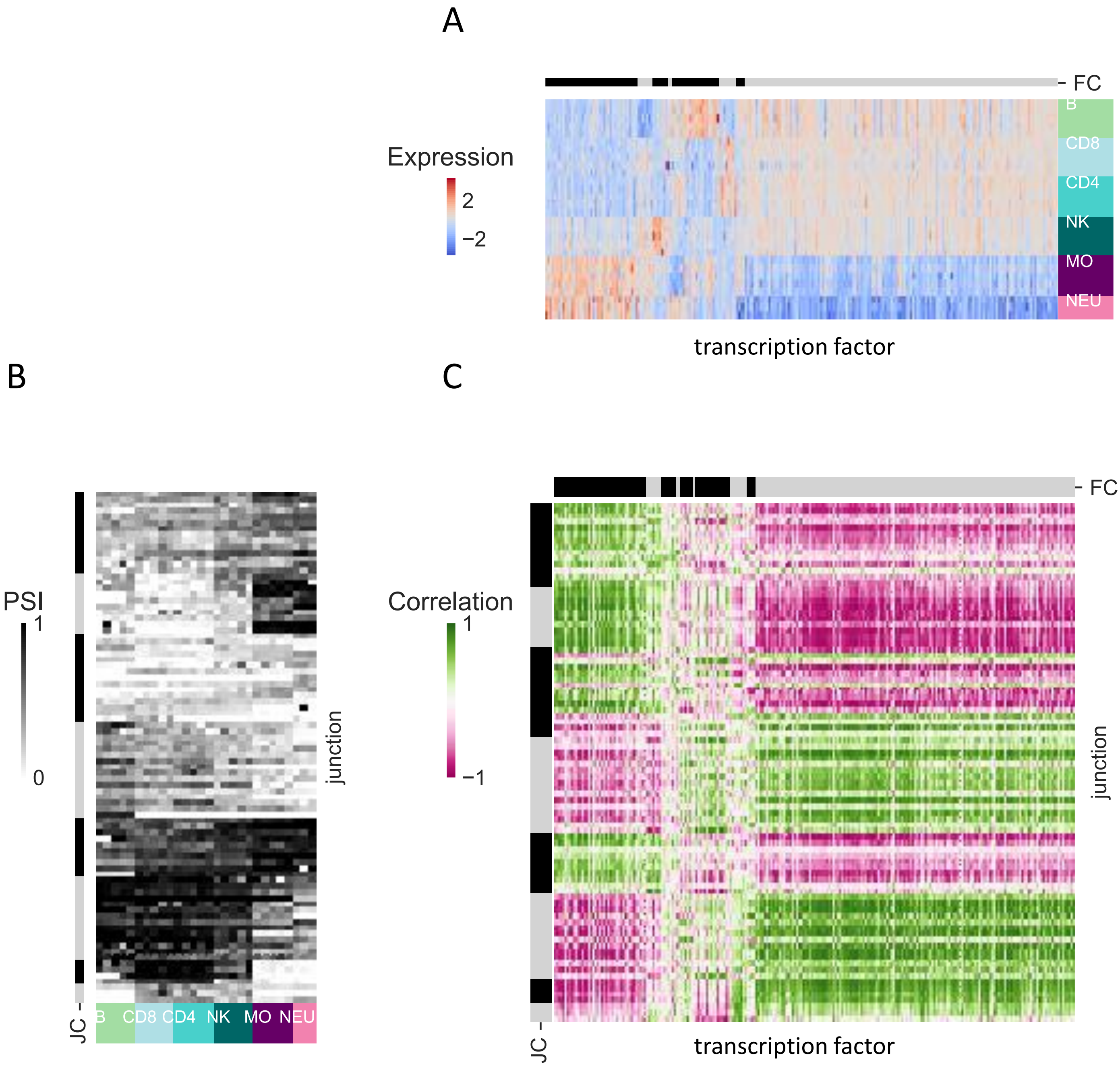
A
FC
B
CD8
CD4
NK
MO
NEU
Expression
2
−2
transcription factor
B
PSI
1
0
JC
B
CD8
CD4
NK
MO
NEU
junction
C
FC
Correlation
1
−1
JC
junction
transcription factor

Figure S10

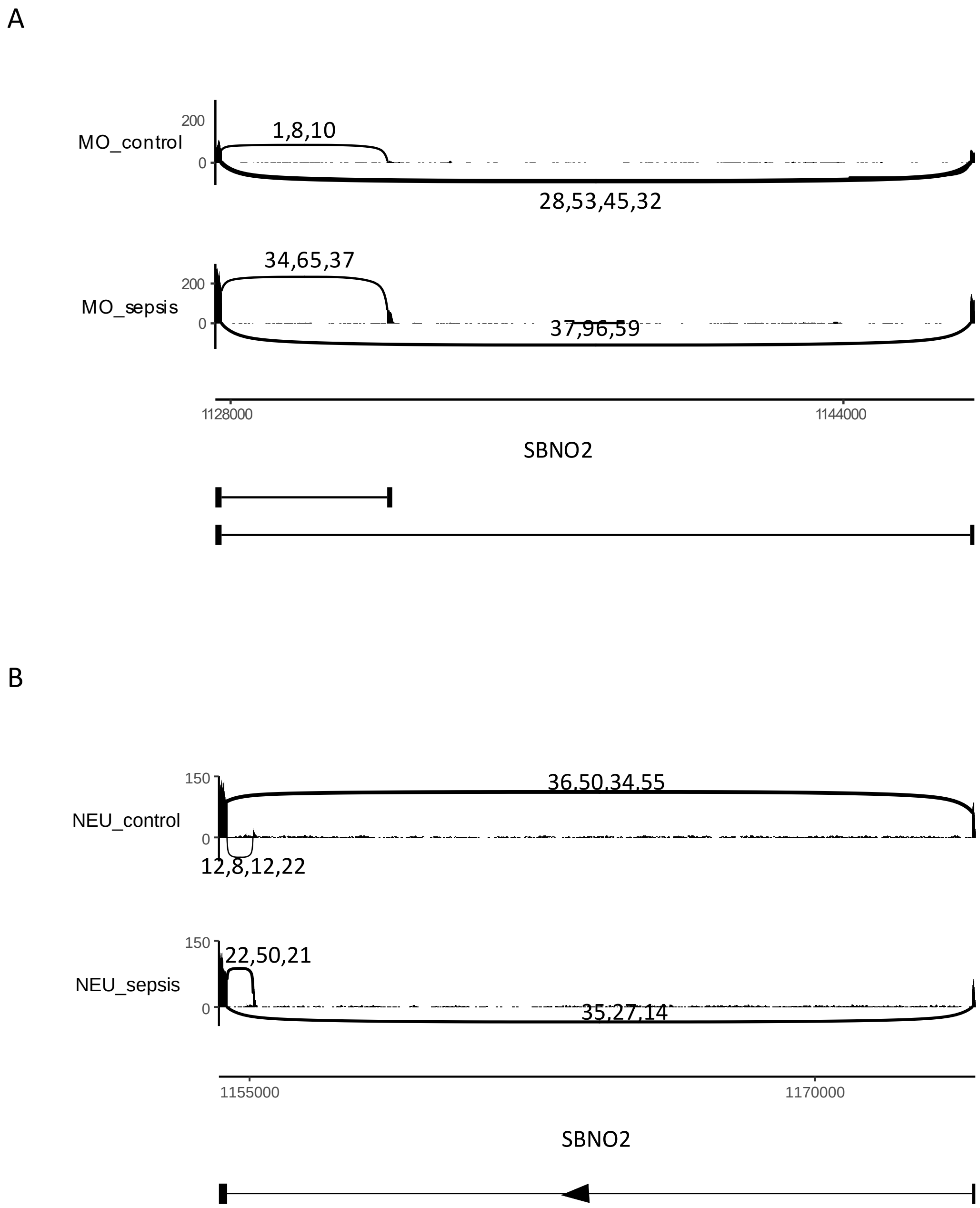

A
MO_control
200
0
1,8,10
28,53,45,32
MO_sepsis
34,65,37
200
0
37,96,59
1128000
1144000
SBNO2
B
NEU_control
150
0
36,50,34,55
12,8,12,22
NEU_sepsis
150
0
22,50,21
35,27,14
1155000
1170000
SBNO2

Figure S11

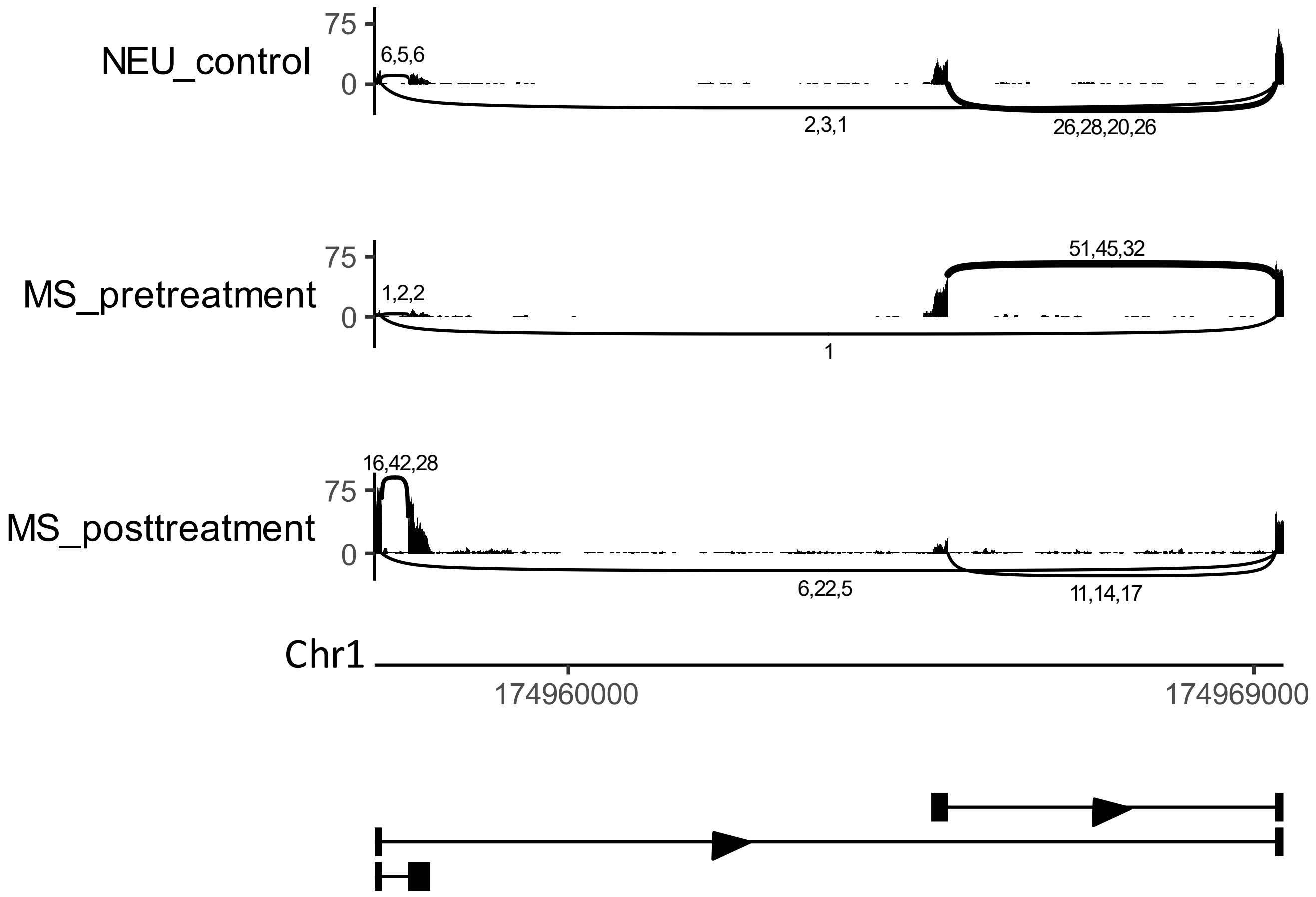

NEU_control
75
0
6,5,6
2,3,1
26,28,20,26
MS_pretreatment
75
0
51,45,32
1,2,2
1
MS_posttreatment
75
0
16,42,28
6,22,5
11,14,17
Chr1
174960000
174969000

Figure S12
